# Factor analysis validates the internal structure of the Cerebellar Neuropsychiatric Rating Scale Version 2 and the five domains of cerebellar neuropsychiatry

**DOI:** 10.3389/fneur.2026.1784525

**Published:** 2026-05-20

**Authors:** Anna L. Burt, Jeremy D. Schmahmann

**Affiliations:** Ataxia Center, Laboratory for Neuroanatomy and Cerebellar Neurobiology, Division of Behavioral Neurology, Department of Neurology, Massachusetts General Hospital and Harvard Medical School, Boston, MA, United States

**Keywords:** behavior rating scale, cerebellar cognitive affective syndrome, cerebellar disease, clinical neurology, neuropsychiatry

## Abstract

**Background:**

The Cerebellar Neuropsychiatric Rating Scale Version 2 (CNRS-2) measures affective symptoms in cerebellar disease patients across five postulated domains of attentional control, emotional control, autism spectrum, psychosis spectrum, and social skill set, each with overshoot and undershoot symptom subdomains. A data-driven approach is needed to test our *a priori* model and to further explore the internal structure of the scale.

**Objectives:**

To explore the latent structure of the CNRS-2 and evaluate the five-domain and overshoot-undershoot structure of the scale items.

**Methods:**

CNRS-2 was administered to 279 cerebellar subjects. Data suitability for factor analysis was assessed. Eigenvalues and parallel analysis scree plots were evaluated for factor extraction. Exploratory factor analysis (EFA) was performed to identify an optimal factor solution. The EFA-derived solution and our *a priori* five-domain model were examined via confirmatory factor analysis (CFA). Fit indices, item loadings, and conceptual interpretability were compared. Bifactor analysis was performed on each of the five domains of the *a priori* model to evaluate whether variance in domain scoring was attributable to the construct measured by the domain versus two subfactors. CFA was also applied to each domain to test the validity of the overshoot-undershoot item groupings.

**Results:**

We extracted 5, 6, and 7 factors based on eigenvalues and parallel analysis. EFA indicated that a six-factor solution best balanced statistical fit and theoretical interpretability. CFA on this EFA-derived solution and on our *a priori* five-domain model showed both to have acceptable statistical fit and internal consistency, but the five-domain model demonstrated stronger conceptual coherence. The bifactor analysis revealed variation in the interpretability of two subfactors within each of the domains, while CFA validated the overshoot-undershoot clustering of symptoms.

**Conclusion:**

Factor analysis of the items comprising CNRS-2 provided empirical support for the clinically derived conceptual framework that defines the five domains of cerebellar neuropsychiatry. Sub-analyses of the individual domains supported the overshoot-undershoot dichotomy. Convergence between our *a priori* domain model and the solutions derived from EFA revealed stable and interpretable symptom clusters. These findings underscore the presence of coherent, multidimensional interrelated neuropsychiatric constructs in cerebellar disease that can be identified and measured by CNRS-2.

## Introduction

The cerebellar cognitive affective/Schmahmann syndrome (CCAS) comprises deficits in executive dysfunction, linguistic processing, spatial cognition, and affect regulation (1–4). The affective component of CCAS has been hypothesized to encompass five neuropsychiatric domains of symptoms including attentional control, emotional control, autism spectrum, psychosis spectrum, and social skill set (5). Additionally, akin to the dysmetria of movement observed in the cerebellar motor syndrome, the neuropsychiatric constructs are conceptualized within a dysmetria of thought hypothesis wherein symptoms are either hypermetric (i.e., exaggerated/positive/overshoot) or hypometric (i.e., diminished/negative/undershoot) (1, 6).

The Cerebellar Neuropsychiatric Rating Scale Version 2 (CNRS-2) provides a comprehensive measure of the neuropsychiatric component of CCAS (7). The CNRS-2 was recently developed as a more refined and rigorously validated version of the original CNRS (8). The 34-item, informant-completed original CNRS was first developed in 2015 via a process of conceptual framework identification and pooling of neuropsychiatric items based upon clinical neurological experience (8). This framework was used to develop the more refined and rigorously validated CNRS-2 (7). Amendments and additions were made to the CNRS items to improve clarity, remove ambiguity, and ensure comprehensibility. A patient-completed, self-report version was also derived to allow the patient’s perspective on their neuropsychiatric state to be captured along with the impression of their informant. The revised scale, in both self- and informant-report versions, was evaluated via cognitive debrief focus groups with cerebellar disease patients and their close family to ensure that all the items in the scale were understandable, relevant, and deemed important to include in the final version of the scale. The final 50-item scale was then psychometrically evaluated in a cohort of 43 cerebellar disease patients and 33 healthy controls, each with a respective nominated informant, such as a parent, spouse, child, or close friend. The scale demonstrated acceptable internal consistency, floor and ceiling effects, and test–retest reliability, and was able to significantly distinguish the cerebellar patients from the healthy controls. Overall, this validation established the CNRS-2 as a valid and reliable measure of neuropsychiatry in cerebellar disease patients (7).

The five domains and overshoot–undershoot dipoles of cerebellar neuropsychiatry were originally conceptualized based on qualitative review of patient, informant, and physician perspective (5). It remains to be shown whether this *a priori* clinical approach can be validated objectively through factor analysis of the CNRS-2 in individuals with cerebellar disorders. We hypothesized that the items that comprise the CNRS-2 would fit into a five-factor structure reflecting the neuropsychiatric domains of *attentional control*, *emotional control*, *autism spectrum*, *psychosis spectrum*, and *social skill set* (1, 5, 7, 8). In our recent psychometric analysis of the CNRS-2 we also evaluated the validity of this domain structure and the overall construct validity of the scale (7). External measures of conceptually related neuropsychiatric constructs were also completed by participants in the validation study to allow analysis of their correspondence to the CNRS-2 domains. There was excellent convergent validity with the external scales indicating that the CNRS-2 provided a reliable overall measure of neuropsychiatric manifestations. However, the CNRS-2 lacked discriminant validity as the five domains correlated well with all of the external measures, whether conceptually related or not, perhaps reflecting the comorbid, interrelated, or overlapping nature of the neuropsychiatric symptoms.

Whereas the development and validation study did not statistically validate the *a priori* five-domain structure of the CNRS-2, qualitative clinical and theoretical insights have supported our five-domain hypothesis. The overlapping and interrelatedness of the neuropsychiatric constructs makes them challenging to clearly separate (7, 9, 10). In the present study, we therefore performed an in-depth, data-driven examination of the CNRS-2 to assess the validity of our *a priori* five-domain structure and the overshoot–undershoot groupings, and to explore other potential factor models for the scale.

## Methods

### Procedures

We recruited adults with a diagnosed cerebellar disorder via advertisements circulated by several ataxia foundations including the National Ataxia Foundation (NAF), Multiple System Atrophy Coalition (Mission MSA), Posterior Fossa Society (PFS), Friedreich’s Ataxia Research Alliance (FARA), Ataxia United Kingdom, the Ataxia Charlevoix-Saguenay Foundation, and the Australian Cerebellar Ataxia Registry, and through patient research invitation letters sent to members of our Massachusetts General Hospital patient list. A small number of participants were recruited through the NAF 2025 Annual Ataxia Conference. All participants were ≥ 18 years old and had at least 6th grade English reading proficiency.

Participants accessed the study via a link included in the advertisement or invitation. The participants recruited at the NAF conference completed paper copies of the study. We then inputted their data into the online questionnaire platform. In lieu of a full (e) Consent, participants reviewed a study information page. They were then asked to complete three online surveys—demographic information, clinical information, and the CNRS-2 questionnaire. This study and its consenting procedure were approved by the Mass General Brigham Institutional Review Board (IRB).

### Materials

#### Demographic and clinical information

Participants were asked to report relevant information including their education length and level, cerebellar disease diagnosis, age of disease onset, first symptom and whether they were concurrently taking any psychiatric medications. They were also asked to rate their ataxia severity according to the Friedreich’s Ataxia Rating Scale (FARS) functional stage, (11) and the Klockgether four-point scale (12) where stage 0 is no gait difficulties; stage 1, impaired gait but independent; stage 2, loss of independent gait (use of cane/walker); and stage 3, wheelchair-dependent.

#### Cerebellar Neuropsychiatric Rating Scale Version 2 (CNRS-2)

The CNRS-2 is a 50-item scale in both self- and informant-report versions. Just the self-report was included in this study. The scale asks the patient to rate the frequency at which they experienced the symptoms measured by the CNRS-2 items over the previous month on a four-point Likert scale (*Never*, *Rarely*, *Sometimes*, *Often*, or *Almost always*). The scale has a maximum score of 200.

Data collection for this study began prior to the publication of the CNRS-2 (7). The order of the CNRS-2 items was revised in the final publication, but we continued to use the original item ordering in this study, rather than switching the order partway through data collection. Supplementary Appendix A is a table mapping the item numbers of the CNRS-2 used in the present study onto the revised numbering of the published scale.

To ensure completeness of the data for statistical analysis, participants who had not completed at least 70% of the CNRS-2 items were excluded from the analysis. Where item responses were missing at random, these participant records were retained with full information maximum likelihood (FIML) used when performing the factor analyses.

### Statistical analysis

**Figure 1:**
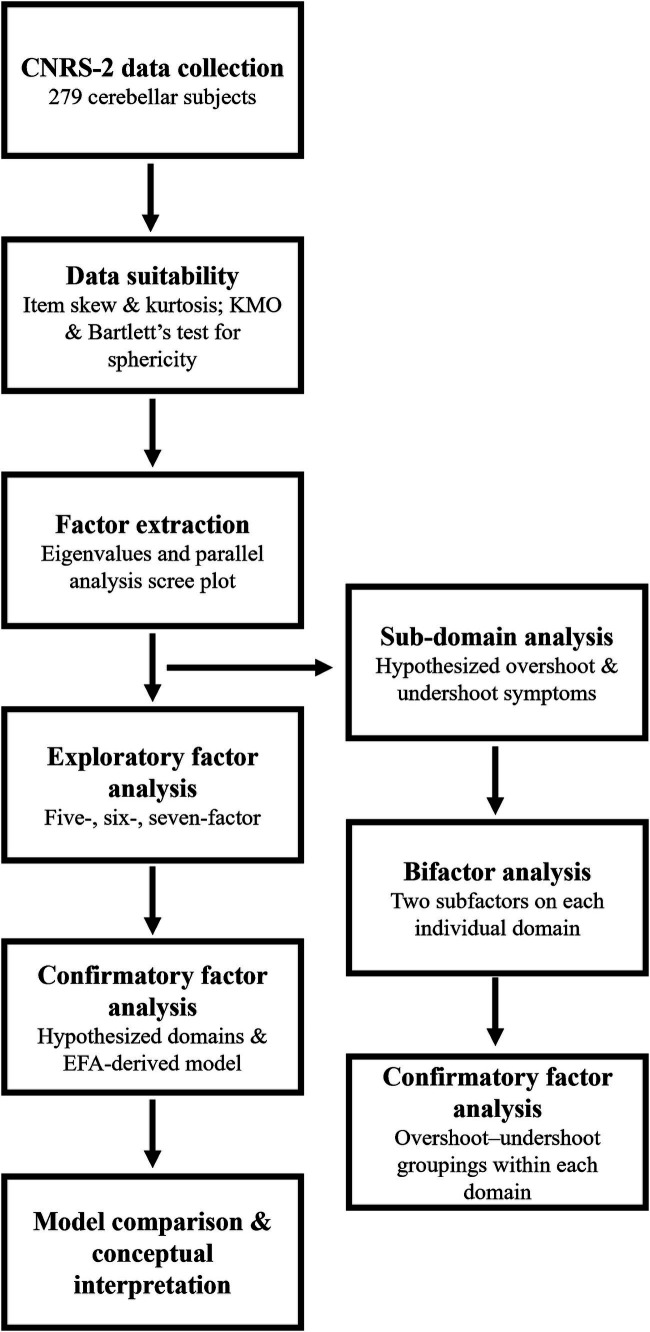
Analytical approach flow chart. The step-by-step approach we adopted to evaluate the factor structure of the CNRS-2 and the subdomain structure of our *a priori* five-domain model. KMO, Kaiser-Meyer-Olkin.

Figure 1 provides a visual representation of our approach to this study.

Analyses were performed in RStudio (13) using the psych (14) package for factor extraction and exploratory factor analysis, and lavaan (15, 16) and semtools (17) packages for the confirmatory factor analyses and bifactor analysis.

We first screened the CNRS-2 data for the effects of demographic and clinical characteristics on participants’ endorsements of the individual CNRS-2 items. We used the Wilcoxon signed rank test to evaluate the influence of participant gender and psychiatric medication use, and the Kruskal-Wallis test for the effects of participants’ cerebellar disorder diagnosis, gait severity, and highest education level. Spearman correlations were evaluated for the impact of participant age, disease duration (i.e., years since disease onset), FARS functional stage, and education duration. We used *p*-values for the individual CNRS-2 items across each demographic or clinical factor, corrected to control for multiple comparisons with the Benjamini-Hochberg procedure.

We then computed descriptive statistics (mean, standard deviation, median, kurtosis, and skewness) for each CNRS-2 item. We considered kurtosis values between + 2 and −2 to be acceptable (18). Items above this range, i.e., many extreme item responses, could destabilize factor analysis; items below this range suggested an item does not discriminate well. We defined acceptable skewness by the same range, with strongly skewed items (> |2|) indicating the presence of floor or ceiling effects (18).

We evaluated the suitability of the item data for factor analysis using the Kaiser-Meyer-Olkin (KMO) measure of sampling adequacy and Bartlett’s test of sphericity. The criteria used for KMO interpretation were KMO values > 0.90 = *marvelous*, 0.80–0.90 = *good*, 0.70–0.80 = *average*, 0.60–0.70 = *mediocre*, 0.50–0.60 = *poor*, and < 0.50 = *unacceptable* (19). Significant Bartlett’s test results (*p* < 0.05) indicated that inter-item correlations were sufficient for factor analysis (20).

We determined the optimal number of factors to extract via (1) Kaiser criterion: eigenvalues for the CNRS-2 data were computed and the eigenvalues greater than one were retained, (21) and (2) parallel analysis: our CNRS-2 eigenvalues were plotted alongside eigenvalues derived from a randomly generated dataset. The factors with eigenvalues exceeding those of the random data were retained, i.e., the factor number where the two eigenvalue lines intersected (22). We considered the result of each method in order to inform the number of factors to extract for the exploratory factor analysis (EFA).

We performed EFA on the CNRS-2 dataset using principal axis factoring with oblique rotation and polychoric correlations. This accounted for the ordinal scale and expected correlations between the latent factors (23).

We interpreted factor loadings (*λ*) of at least 0.30 as acceptable (24) and communalities (*h^2^*) > 0.50 were considered good (25). We evaluated the acceptability of the EFA solutions according to the following fit indices thresholds (26): root mean square error of approximation (RMSEA) < 0.05; Tucker-Lewis index (TLI) > 0.90; and standardized root mean squared residual (SRMR) < 0.08. We also compared chi-squared (*χ^2^*) values and Bayesian information criterion (BIC) values between the factor solutions with smaller values considered better. Robust (scaled) fit indices are reported in our results due to our ordinal, non-normal dataset. Qualitative conceptual interpretation was also performed to ensure that the solutions derived from the EFA were coherent, aligned with neuropsychiatric theory, and held utility for use in clinical settings. We determined an optimal EFA-derived factor solution by considering both the strength of statistical fit and conceptual interpretability.

We performed confirmatory factor analysis (CFA) in the same dataset, employing the diagonally weighted least squares with mean and variance adjustment (WLSMV) estimator. We evaluated our five-domain *a priori* model (*attentional control*, *emotional control*, *autism spectrum*, *psychosis spectrum*, and *social skill set* as factors) and the optimal EFA-derived solution.

CFA loadings (*λ*) and statistical significance were evaluated and compared between the models. We used the same fit indices criteria as in the EFA in addition to the comparative fit index (CFI), with > 0.90 considered a good fit (26). We also evaluated reliability and internal consistency for each factor using Cronbach’s alpha (*α*), with values > 0.70 considered acceptable and > 0.90 considered excellent (27–29). We evaluated convergent validity using average variance extracted (AVE), with AVE > 0.50 indicating good convergence of the items within the factor (29).

We performed sub-analyses within the domains of the *a priori* five-domain model to evaluate the presence of two subdomains of dichotomous overshoot and undershoot symptoms. First, we used bifactor analysis to determine whether variance in scoring within each domain was more attributable to an overall general factor, i.e., the neuropsychiatric construct purported to be measured by the domain, or to two separate subfactors within the domain. Omega hierarchical (ω_h_) values > 0.70 were interpreted as suggesting variance in scores was predominantly influenced by the overall general factor, while values < 0.50 indicated that the subfactors may be more meaningful (30–32). We then performed CFA on each of the five domains to evaluate our *a priori* groupings of items into the overshoot and undershoot subdomains for each of the domains.

## Results

### Participant characteristics

Our study cohort comprised 279 patients with a diagnosed cerebellar disorder. Forty-five different cerebellar diagnoses were reported, thus reflecting a heterogeneous cohort, with spinocerebellar ataxia (SCA) type 3 (*n* = 47), SCA6 (*n* = 38), and SCA27B (*n* = 24) the most common. The full diagnostic composition of the cohort is shown in Table 1.

**Table 1:** Cohort diagnoses.

Diagnosis			
Acquired ataxia	1	SCA6	37
Ataxia with oculomotor apraxia type 2 (AOA2)	2	SCA7	3
Autosomal recessive cerebellar ataxia type 1 (ARCA1)	3	SCA8	17
Autosomal recessive spastic ataxia of charlevoix-saguenay (ARSACS)	6	SCA10	3
Cerebellar ataxia with neuropathy and vestibular areflexia syndrome (CANVAS)	12	SCA12	2
Episodic ataxia type 2 (EA2)	5	SCA13	1
Friedreich ataxia (FA)	7	SCA14	3
Head injury	1	SCA15	2
Hereditary spastic paraplegia (HSP)	3	SCA19/22	1
Hypertrophic olivary degeneration (HOD)	1	SCA20	1
Idiopathic ataxia	3	SCA27B	24
Immune-mediated ataxia	5	SCA28	3
Multiple system atrophy (cerebellar type) (MSA-C)	2	SCA33	1
Palatal tremor	1	SCA34	3
Paraneoplastic cerebellar degeneration	1	SCA35	1
Posterior fossa syndrome	4	SCA36	2
*POLR3* mutation	1	SCA42	4
Spinocerebellar ataxia (SCA) type unknown	8	SCA48	2
SCA1	16	SCA50	1
SCA2	13	*SCN1A* mutation	1
SCA3	47	Unknown cause of cerebellar ataxia	14
SCA4	1	Unspecified cerebellar ataxia	9
SCA5	1		

Most participants were from the United States (65.6%), the mean participant age was 58.0 years (SD 15.7), and mean duration of cerebellar disease was 14.0 years (SD 11.9). The mean Friedreich’s Ataxia Rating Scale (FARS) functional stage was 3.01 (SD 1.25) indicating that on average participants experienced mild motor disability requiring use of a cane or walker for stability and walking. Concurrent use of at least one psychiatric medication was reported by 41.6% of participants. Further demographic and clinical information are shown in Table 2. For the overall CNRS-2 assessment, the mean total score was 41.32 (SD 29.96), median was 36, and minimum and maximum scores were 0 and 136, respectively.

**Table 2:** Cohort characteristics.

Demographic information		
*N*	279	
Mean (SD) age in years	58.0 (15.7)	
Gender	58.1% female	
Mean (SD) years of education	14.5 (4.5)	
Country, *n* (% of total cohort)	United States	183 (65.6%)
	Australia	65 (23.3%)
	Canada	9 (3.2%)
	United Kingdom	5 (1.8%)
	Brazil	4 (1.4%)
	India	3 (1.1%)
	New Zealand	2 (0.7%)
	Chile	1 (0.4%)
	France	1 (0.4%)
	Peru	1 (0.4%)
	Philippines	1 (0.4%)
	Poland	1 (0.4%)
	Singapore	1 (0.4%)
	Slovenia	1 (0.4%)
	Spain	1 (0.4%)
	United Arab Emirates	1 (0.4%)

Clinical information		
Mean (SD) age of onset of ataxia symptoms	44.0 (19.0)	
Mean (SD) duration of disease in years	14.0 (11.9)	
Mean (SD) FARS Functional Stage (0–6)	3.01 (1.25)	
Gait severity, *n* (% of total cohort)	0: Normal gait/walking	17 (6.1%)
	1: Some gait/walking problems but independent	116 (41.6%)
	2: Require use of walking aid	116 (41.6%)
	3: Use of wheelchair	30 (10.8%)
Use of a psychiatric medication	41.6% yes	
CNRS-2 total score	Mean (SD)	41.32 (29.96)
	Median	36
	Range	0–136

Screening of the dataset for demographic and clinical effects revealed that motor ataxia severity, quantified via patient-reported functional stage and gait severity measures, did not significantly affect CNRS-2 item scoring, adjusted *p* > 0.05 for both measures across all CNRS-2 items. Similarly, education level and education duration had no effect on item scores, and cerebellar diagnosis and disease duration also did not significantly influence scoring. Age and gender seemed to have an effect on the scoring of some items, *p* < 0.05, however there were no significant effects in the majority of cases. There were significant associations between participant use of psychiatric medication and scores for most items, *p* < 0.05. The mean total CNRS-2 score for participants reporting use of a psychiatric medication was significantly higher than participants who did not report use of a medication, compared via the Mann–Whitney *U* test, *p* < 0.001, mean total scores = 52.7 (SD 32.9) and 32.4 (SD 24.4), respectively. See Supplementary Table S1 for the full screening results.

### CNRS-2 item descriptive statistics

Descriptive statistics for each of the 50 CNRS-2 items, including mean (SD), median, skewness, and kurtosis, are shown in Table 3. Mean skewness across the 50 items was 1.33 (SD 0.80), with items 2 (3.90; “*I experience hallucinations, or see or hear things that others do not*”) and 32 (3.63; “*I make repetitive movements or sounds that follow a particular pattern*”) showing the greatest skewness and item 3 (0.14; “*I feel indifferent, uninterested, or unmotivated*”) the least. Mean kurtosis was 1.77 (SD 3.50), with the same items at each extreme; 16.94 and 13.81 for items 2 and 32, respectively, and −0.94 for item 3. The overall skewness and kurtosis for the scale suggested that the data were suitable for factor analysis, despite some item-level deviations from normality.

**Table 3:** CNRS-2 item descriptive statistics.

CNRS-2 item	Mean score	SD	Median	Skew	Kurtosis
1	1.437	1.0048	1	0.320	−0.427
2	0.168	0.5467	0	3.898	16.941
3	1.441	1.0607	1	0.136	−0.940
4	0.785	1.0913	0	1.275	0.672
5	0.416	0.8218	0	2.168	4.468
6	0.441	0.8624	0	2.244	4.756
7	0.910	1.0842	1	1.122	0.487
8	0.928	1.1325	0	0.925	−0.258
9	0.878	1.0625	1	1.085	0.372
10	0.262	0.6676	0	2.756	7.094
11	0.584	0.8891	0	1.398	1.036
12	0.703	0.9259	0	1.188	0.528
13	0.484	0.9628	0	2.142	3.881
14	1.222	1.1908	1	0.624	−0.617
15	0.692	1.0063	0	1.402	1.154
16	1.118	1.1365	1	0.779	−0.212
17	1.384	1.0993	1	0.401	−0.668
18	1.351	1.2632	1	0.474	−0.957
19	0.968	1.1200	1	0.936	−0.107
20	0.480	0.7994	0	1.580	1.571
21	0.523	0.9008	0	1.725	2.301
22	0.857	1.0598	0	1.099	0.400
23	0.337	0.6579	0	2.312	6.161
24	0.366	0.6535	0	1.929	3.688
25	0.814	1.0041	1	1.227	0.841
26	0.896	1.0795	1	1.061	0.179
27	0.932	1.0589	1	0.950	0.062
28	0.964	1.0823	1	0.885	−0.220
29	1.168	1.1011	1	0.648	−0.443
30	1.406	1.2123	1	0.533	−0.707
31	1.201	1.1631	1	0.648	−0.559
32	0.244	0.7233	0	3.634	13.807
33	1.151	1.1331	1	0.741	−0.356
34	1.449	1.1852	1	0.440	−0.695
35	0.612	0.9487	0	1.676	2.313
36	0.641	0.8852	0	1.231	0.813
37	1.033	1.0966	1	0.842	−0.142
38	0.348	0.7400	0	2.246	4.648
39	0.366	0.8046	0	2.546	6.537
40	0.714	0.9504	0	1.278	1.058
41	0.402	0.7443	0	1.996	3.804
42	1.094	1.0678	1	0.777	−0.032
43	1.051	1.0536	1	0.866	0.189
44	1.120	1.1553	1	0.824	−0.186
45	0.540	0.8667	0	1.631	2.096
46	0.917	1.0394	1	0.882	−0.151
47	0.489	0.8112	0	1.745	2.592
48	1.330	1.2284	1	0.599	−0.622
49	0.902	1.1027	1	1.133	0.475
50	0.667	0.9598	0	1.514	1.886

### Data suitability

The overall KMO value was 0.94, exceeding the recommended minimum of 0.60 and therefore indicating that the items were well correlated and had *marvelous* (19) sampling adequacy. Bartlett’s test of sphericity also showed that items were significantly correlated, *χ^2^* (1225) = 8878.7, *p* < 0.001. The KMO values for the individual items are shown in Supplementary Table S2. These tests indicated that the data were suitable for factor analysis.

### Factor extraction

**Figure 2:**
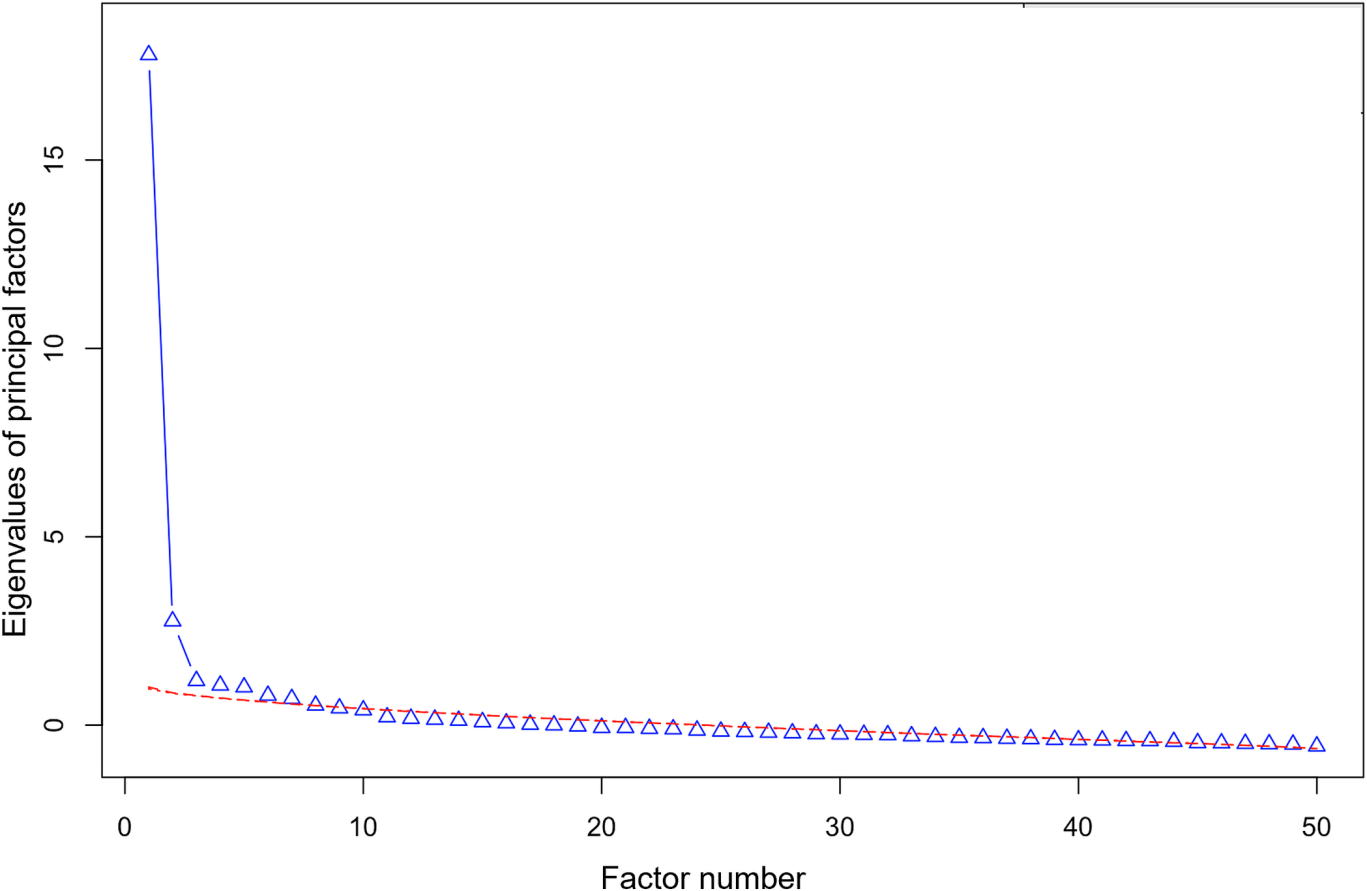
Parallel analysis scree plot. Parallel analysis scree plot to determine the number of factors to extract and retain for EFA. Our CNRS-2 data eigenvalues (triangles, in blue) are plotted against eigenvalues for random simulated and resampled data (dashed lines, in red). The point where our blue eigenvalue line crosses the red simulated data line indicates the number of factors that should be retained for EFA, i.e., when the CNRS-2 eigenvalue is greater than the simulated eigenvalue.

Using the Kaiser method, the CNRS-2 eigenvalues indicated that five factors should be considered for extraction (factor number 5 *λ* = 1.008), presented in Supplementary Table S3. The parallel analysis showed our plotted CNRS-2 eigenvalue line converging with the simulated data line at factor number 7 (Figure 2). We decided to perform the exploratory factor analysis (EFA) on five, six, and seven-factor solutions to account for variation in interpretation between the two factor retention methods.

### Exploratory factor analysis (EFA)

#### Five-factor solution

The five-factor solution demonstrated the smallest item complexity, 2.1, and BIC, −3512.64, indicating that it was the most parsimonious model. Additionally, for this solution, only 3 of the 50 items had a factor loading less than 0.30. However, the fit indices for this solution were high relative to the other factor solutions: RMSEA = 0.062 [90% CI: 0.058–0.066], TLI = 0.827, and SRMR = 0.05. Factor 1 comprised 17 items with factor loadings from 0.35–0.73. Factor 2 comprised 9 items with loadings from 0.32–0.90. Factor 3 comprised 19 items with loadings from 0.23–0.73. Factor 4 comprised 5 items with loadings from 0.37–0.57. No items meaningfully loaded onto the fifth factor. Mean item communality (*h^2^*) was 0.49 (range = 0.22–0.78). Item loadings are shown in Supplementary Table S4; Supplementary Figure S1 is a path diagram reflecting the five-factor EFA solution.

#### Six-factor solution

The six-factor solution had higher mean item complexity, 2.4, and higher BIC, −3478.51. However, overall, it was a relatively strong statistical fit: RMSEA = 0.058 [90% CI: 0.054–0.062], SRMR = 0.04, and TLI = 0.849—just shy of the “good” threshold. There were some items with loadings below 0.30 and several instances of cross-loadings, however all items had a loading of at least 0.25. Factor 1 comprised 15 items with loadings from 0.25–0.72. Factor 2 comprised 7 items with loadings from 0.44–0.91. Factor 3 comprised 13 items with loadings from 0.25–0.63. Factor 4 comprised 5 items with loadings from 0.29–0.59. Factor 5 comprised 4 items with loadings from 0.34–0.40. And factor 6 comprised 6 items with loadings from 0.32–0.71. Mean item h^2^ was 0.52 (range = 0.22–0.80). The six factors were conceptually interpretable. Item loadings are shown in Supplementary Table S5; Supplementary Figure S2 is a path diagram reflecting the six-factor EFA solution.

#### Seven-factor solution

The seven-factor solution had the best statistical fit of the three solutions with the strongest fit indices: RMSEA = 0.053 [90% CI: 0.049–0.058], TLI = 0.871, and SRMR = 0.04. However, BIC was high relative to the other solutions reflecting the increased complexity added by retaining more factors (BIC = −3436.41). Factor 1 comprised 9 items with loadings from 0.28–0.73. Factor 2 comprised 9 items with loadings from 0.33–0.90. Factor 3 comprised 12 items with loadings from 0.24–0.58. Factor 4 comprised 5 items with loadings from 0.29–0.67. Factor 5 comprised 2 items with loadings of 0.53 and 0.60. Factor 6 comprised 5 items with loadings from 0.32–0.69. And factor 7 comprised 8 items with loadings from 0.31–0.57. Mean item h^2^ was 0.54 (range = 0.22–0.81). Conceptually the seven-factor solution was not easily interpretable with the fourth, fifth, and sixth factors having relatively few items, several cross-loadings, and several items not exceeding the loading threshold of 0.30. Item loadings are shown in Supplementary Table S6; Supplementary Figure S3 is a path diagram reflecting the seven-factor EFA solution.

The statistical fit indices are shown for the three models in Table 4. We determined that the six-factor solution provided the best balance of statistical fit and conceptual interpretability, so we selected this EFA-derived model for further examination via CFA. Further references to the factors of the six-factor solution will use the labels *F1, F2, etc.*

**Table 4:** Fit indices for EFA factor solutions.

Fit index	Five-factor	Six-factor	Seven-factor
Mean item complexity	2.1	2.4	2.5
Null model objective function (df)	34.26 (1225)	34.26 (1225)	34.26 (1225)
Objective function (df)	7.96 (985)	7.12 (940)	6.33 (896)
Likelihood *χ^2^* (*p*-value)	2023.45 (<0.001)	1804.66 (<0.001)	1599.47 (<0.001)
RMSEA	0.062 [0.058–0.066]	0.058 [0.054–0.062]	0.053 [0.049–0.058]
BIC	−3512.64	−3478.51	−3436.41
TLI	0.827	0.849	0.871
SRMR	0.05	0.04	0.04

df, degrees of freedom; *χ^2^*, chi-squared; RMSEA, root mean square error of approximation; BIC, Bayesian information criterion; TLI, Tucker-Lewis index; SRMR, standardized root mean squared residual.

### Confirmatory factor analysis (CFA)

#### EFA-derived six-factor solution

CFA on the EFA-derived six-factor solution demonstrated strong statistical fit: RMSEA = 0.053 [90% CI: 0.049–0.057], CFI = 0.941, TLI = 0.937, and SRMR = 0.078. Item loadings were strong with all items above the threshold of 0.40 (range = 0.560–0.940), the items with the lowest loadings were item 25 (“*I am overly trusting of others*”; *λ* = 0.596), item 37 (“*I am overly sensitive to people entering my personal space*”; λ = 0.560), item 38 (“*I cry and/or laugh in an exaggerated or inappropriate way or without an apparent trigger*”; λ = 0.595), and item 2 (“*I experience hallucinations, or see or hear things that others do not*”; λ = 0.600; Supplementary Table S7). Internal consistency was at least acceptable across all six factors: Cronbach *α* > 0.90 for *F1*, *F2*, and *F3*, and Cronbach α > 0.70 for *F4*, *F5*, and *F6*. AVE was greater than or equal to 0.50 for all factors indicating good convergent validity of the items within each factor. Correlations between the domains were moderate-to-good, indicating variable discriminant validity. The weakest correlation was between *F2* and *F4* (*ρ* = 0.57), and the strongest was between *F1* and *F3* (*ρ* = 0.88; Supplementary Table S8).

#### Five-domain *a priori* model

CFA on our *a priori* five-domain model demonstrated an acceptable statistical fit: *χ^2^* (df) = 2457.1 (1165), CFI = 0.915, TLI = 0.910, RMSEA = 0.064 [90% CI: 0.060–0.067], SRMR = 0.091. All items had loadings greater than 0.40 (Supplementary Table S9), with standardized loadings ranging from 0.516–0.903. The structure was reliable, with internal consistency at least acceptable across all of the domains: Cronbach *α* > 0.90 for *emotional control*, *attentional control*, and *social skill set*, and Cronbach *α* > 0.70 for *psychosis spectrum* and *autism spectrum*. *Attentional control*, *emotional control*, and *social skill set* also had good convergent validity, AVE > 0.50, however *autism spectrum* and *psychosis spectrum* did not meet this threshold. Correlations between the domains were high, with the lowest correlation between *emotional control* and *autism spectrum* (*ρ* = 0.77) and the strongest between *attentional control* and *autism spectrum* (*ρ* = 0.97), suggesting weak discriminant validity (Supplementary Table S10).

**Figure 3:**
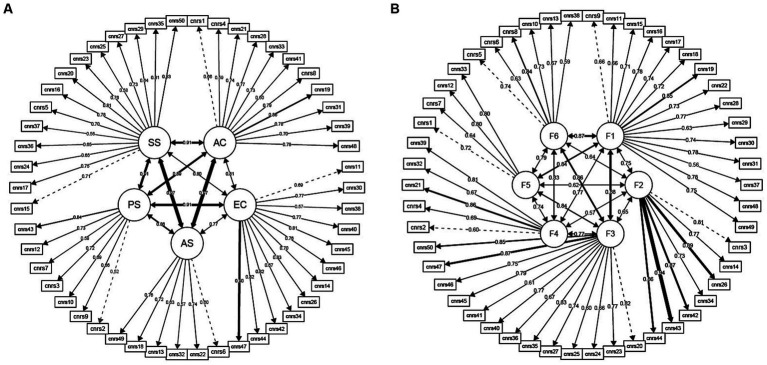
Confirmatory factor analysis path diagrams. Path diagrams showing the CFA loadings of the CNRS-2 items onto their respective factors and the correlations between the factors themselves. The thickness of the line corresponds to the strength of the loading or correlation, i.e., thicker line between item and factor indicates a stronger loading, while a thicker line between factors indicates a higher correlation coefficient. **(A)** *A priori* five-domain model and **(B)** EFA-derived six-factor solution. AC, attentional control; EC, emotional control; AS, autism spectrum; PS, psychosis spectrum; SS, social skill set.

The fit indices for the two models are shown in Table 5 and reliability statistics in Table 6. Figure 3 shows path diagrams for the CFA loadings for Figure 3A the a priori five domains and Figure 3B the EFA-derived six-factors. Overall, the EFA-derived six-factor solution had a stronger statistical fit. Both solutions had strong item loadings and acceptable-to-excellent reliability.

**Table 5:** Fit indices for CFA on the EFA-derived solution and *a priori* model.

Fit index	EFA-derived six-factor	Hypothesized five-factor
*χ*^2^(df)	2061.35 (1160)	2457.06 (1165)
RMSEA	0.053 [0.049–0.057]	0.064 [0.060–0.067]
CFI	0.941	0.915
TLI	0.937	0.910
SRMR	0.078	0.091

*χ^2^*, chi-squared; df, degrees of freedom; RMSEA, root mean square error of approximation; CFI, comparative fit index; TLI, Tucker-lewis index; SRMR, standardized root mean squared residual.

**Table 6:** Internal consistency of the EFA-derived solution and *a priori* model.

EFA-derived six-factor solution			Hypothesized five-domain solution		
Factor number	Cronbach α [CI]	AVE	Domain	Cronbach α [CI]	AVE
F1	0.92 [0.91–0.93]	0.53	Attentional control	0.89 [0.87–0.91]	0.54
F2	0.92 [0.90–0.93]	0.71	Emotional control	0.90 [0.88–0.92]	0.58
F3	0.91 [0.89–0.92]	0.57	Autism spectrum	0.74 [0.69–0.79]	0.45
F4	0.74 [0.68–0.78]	0.53	Psychosis spectrum	0.77 [0.73–0.81]	0.47
F5	0.77 [0.71–0.80]	0.55	Social skill set	0.90 [0.87–0.91]	0.51
F6	0.75 [0.70–0.79]	0.50			

CI, confidence interval; AVE, average variance extracted.

### Conceptual interpretation

The organization of the CNRS-2 items into the EFA-derived six-factor solution is shown in Supplementary Appendix B, and the *a priori* five-domain model is shown in Supplementary Appendix C, with the CFA loading (*λ*) listed for each item.

The EFA-derived six-factor model showed six conceptually interpretable neuropsychiatric constructs in the clustering of items within the factors. *F1* reflects symptoms of *anxiety and cognitive rigidity*. *F2* reflects a very coherent *depressive* construct. *F3* reflects a measure of *social–emotional disinhibition*, comprising items measuring impulsive or inappropriate social behavior, lack of awareness of social cues and boundaries, and mismatched emotional responses to others and situations. If item 2, concerning hallucinations and with a relatively lower loading, is excluded from *F4* then a more interpretable construct of *repetitive and hyperactive behavior* becomes clear. *F5* comprises only four items, however these seem to reflect an *attentional and emotional under-responsiveness* including flattened affect and inattention. *F6*, while heterogenous, overall reflects *emotional and sensory dysregulation* encompassing emotional lability, intrusive or obsessive thoughts, and exaggerated response to sensory stimulation.

### Evaluation of overshoot–undershoot subdomain structure

#### Bifactor analysis

**Figure 4:**
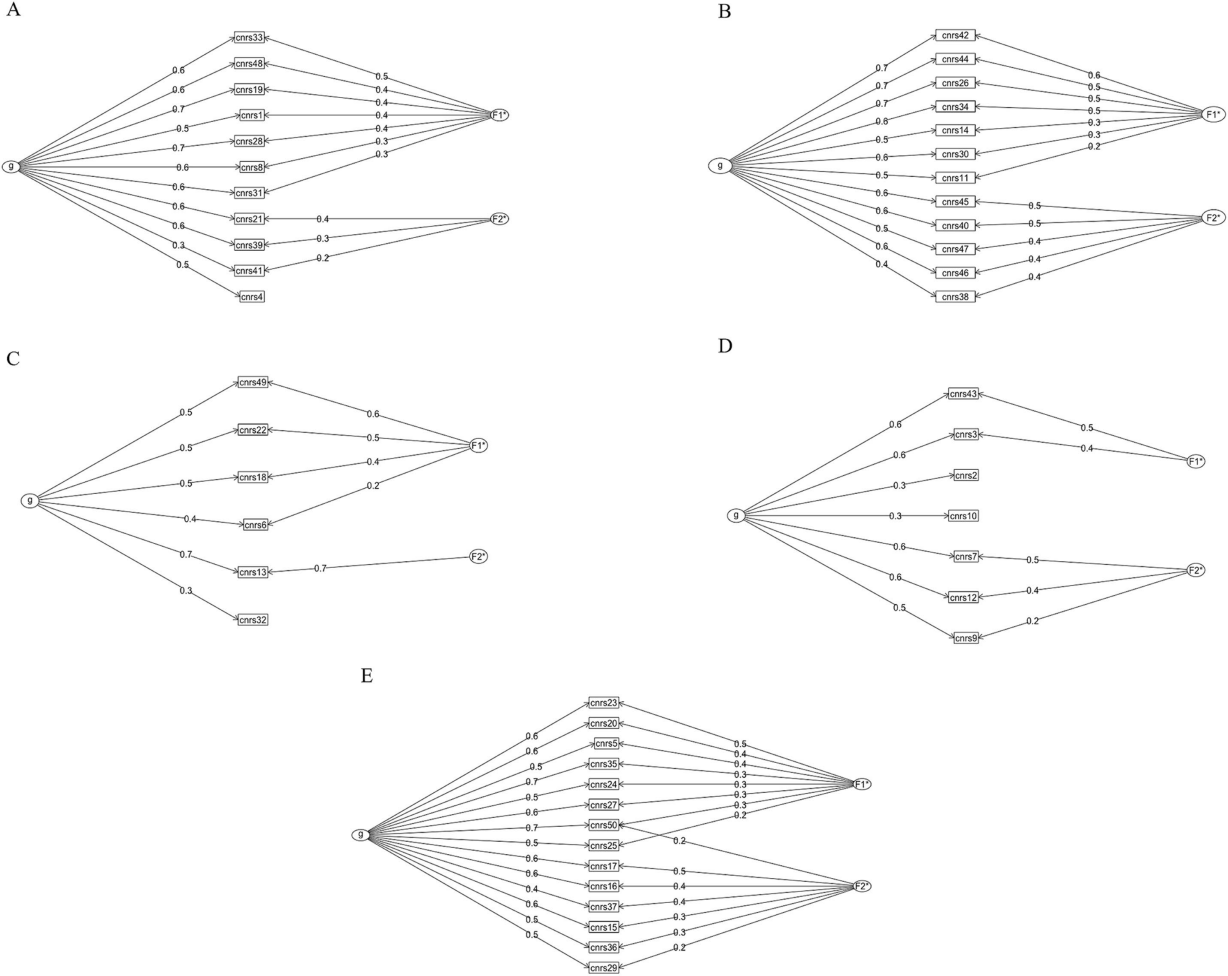
Bifactor path diagrams for each of the five *a priori* domains. Path diagrams visualizing the bifactor analysis performed on each of the five *a priori* domains to evaluate the effect of the overall neuropsychiatric construct (g) measured by the domain on CNRS-2 scores and the variation attributable to two subdomains within the construct. **(A)** *Attentional control*, **(B)** *emotional control*, **(C)** *autism spectrum*, **(D)** *psychosis spectrum*, and **(E)** *social skill set*.

We performed bifactor analyses on each of the five domains of our *a priori* model to evaluate whether the overarching neuropsychiatric construct measured by the domain was the dominant influential factor, or if variation was attributable to multiple subdomains. For the *attentional control* and *social skill set* domains, most variation was attributable to the overall constructs measured by the domains with less influence from subfactors, *ω_h_* = 0.70 and 0.71, and ECV = 0.72 and 0.70, respectively. The *emotional control*, *autism spectrum*, and *psychosis spectrum* domains had more interpretable subfactors (*emotional control*: *ω_h_* = 0.69, ECV = 0.63; *autism spectrum*: *ω_h_* = 0.49, ECV = 0.49; and *psychosis spectrum*: ω_h_ = 0.62, ECV = 0.67). Omega results from the bifactor analysis are shown in Table 7. Figure 4 includes bifactor path diagrams for each of the (A) *attentional control*, (B) *emotional control*, (C) *autism spectrum*, (D) *psychosis spectrum*, and (E) *social skill set* domains.

**Table 7:** Domain bifactor analysis.

Domain	ω_h_	ω_t_	Cronbach α (GLB)	ECV	SRMR	RMSEA	BIC	*χ^2^*	*p*-value
Attentional control	0.72	0.90	0.89 (0.89)	0.72	0.05	0.078 [0.060–0.098]	−99.27	91.83	<0.001
Emotional control	0.69	0.92	0.90 (0.92)	0.63	0.05	0.092 [0.076–0.109]	−98.62	143.05	<0.001
Autism spectrum	0.49	0.79	0.74 (0.72)	0.49	0.04	0.000 [0.000–0.087]	−18.88	3.6	0.46
Psychosis spectrum	0.62	0.80	0.77 (0.77)	0.67	0.07	0.068 [0.026–0.110]	−26.73	18.24	0.02
Social skill set	0.71	0.91	0.90 (0.91)	0.70	0.07	0.096 [0.083–0.110]	−132.12	227.58	<0.001

df, degrees of freedom; ω_h_, omega hierarchical; ω_t_, omega total; GLB, greater lower bound; ECV, explained common variance; SRMR, standardized root mean squared residual; RMSEA, root mean square error of approximation; BIC, Bayesian information criterion; *χ^2^*, chi-squared.

#### Confirmatory factor analysis (CFA)

CFA was applied separately to each of the hypothesized domains with our *a priori* two subfactor overshoot and undershoot groupings of the CNRS-2 items. SRMR was good, < 0.070, for all domains aside from *emotional control,* SRMR = 0.092 (Supplementary Table S11). However, RMSEA was poor for all domains, although approaching acceptability for *attentional control* and *social skill set*, RMSEA = 0.081 and 0.084, respectively. The incremental fit indices were good across all the domains, CFI ≥ 0.943 and TLI ≥ 0.924. Item loadings on the subdomains were strong (*λ* > 0.40; Supplementary Tables S12–S16). For *attentional control overshoot*, loadings ranged 0.498–0.818 and *undershoot* 0.705–0.875. For *emotional control overshoot* 0.534–0.846 and *undershoot* 0.645–0.915. For *autism spectrum overshoot* 0.533–0.725 and *undershoot* 0.673–0.737. For *psychosis spectrum overshoot* 0.598–0.719 and *undershoot* 0.701–0.799. For *social skill set overshoot* 0.587–0.782 and *undershoot* 0.588–0.845. Reliability and internal consistency were strong (Cronbach *α* > 0.80 and AVE > 0.50) across the subdomains, except for *autism spectrum overshoot* and *undershoot*, and *psychosis spectrum overshoot* where Cronbach *α* < 0.70 and AVE < 0.50 (Supplementary Table S17).

## Discussion

In this study, our primary aim was to evaluate the latent structure of the CNRS-2 by exploring empirically derived factor solutions, to test the validity of our *a priori* five-domain structure, and to compare and contrast potential alternative factor solutions. Additionally, we sought to explore the presence of dysmetria in the neuropsychiatric symptom manifestations measured by the scale, determining whether each of our hypothesized domains comprised two subdomains of overshoot and undershoot symptoms. By integrating exploratory and confirmatory factor analyses with conceptual interpretation, we adopted a complementary approach to evaluating the factor structure of the CNRS-2.

The exploratory analysis on five, six, and seven-factor solutions revealed the inherent challenge in separating highly interrelated neuropsychiatric symptoms into distinct conceptual constructs. The five-factor model offered the simplest solution, but no item loaded sufficiently onto the fifth factor. The seven-factor model was the strongest statistical fit for our CNRS-2 dataset; however, it also lacked conceptual interpretability despite its meaningful loadings, strong fit indices, and reliability. Overall, the six-factor solution provided the best balance of statistical fit, parsimony, and interpretability. Conceptual coherence and interpretability was an important aspect to consider and weigh up against statistical fit for each model. This ensures that the factor structure is both statistically sound and has functional utility for scale interpretation when completed by patients in clinical settings for informing their care, management, and treatment.

Importantly, coherent and interpretable constructs were observed for each of the factors of the EFA-derived six-factor model. These constructs showed some alignment with constructs measured by the five domains of our *a priori* model. The *emotional control* domain of our hypothesized model shows overlap with *F1* and *F2* of the six-factor solution, measuring anxiety and cognitive rigidity, and depressive symptoms, respectively. The *social skill set* domain mirrors the behaviors measured in *F3* and *F5*, capturing social–emotional disinhibition and under-responsiveness, respectively.

Confirmatory factor analysis demonstrated that the *a priori* five-domain structure had adequate statistical fit, with good reliability and convergent validity. This supported the conceptual framework that guided the development of the scale, derived from clinical experience and insight and reflecting the five neuropsychiatric constructs conceptualized as the affective component of the CCAS (1, 5). Our analysis provides empirical support for this theoretically driven model.

Bifactor analysis within each domain of the *a priori* model revealed that variability in domain scoring was somewhat attributable to subfactor within the domains of *emotional control*, *autism spectrum*, and *psychosis spectrum*. The subsequent CFA testing the *a priori* subfactor structures of overshoot and undershoot symptoms within each domain yielded acceptable items loadings across all domains, and good reliability in all cases except for *autism spectrum* overshoot and undershoot, and *psychosis spectrum* overshoot. However, there was variability across the domains in the strength of statistical fit indices. This sub-analysis provides some support for our *a priori* overshoot–undershoot groupings of the CNRS-2 items. The weak statistical fit revealed by the CFA and the results of the bifactor analyses underscore the challenge of separating such closely related and overlapping items within a single conceptual construct.

Convergence between the *a priori* model and EFA-derived six-factor model provides evidence for the validity of core neuropsychiatric features conceptualized within the affective component of the cerebellar cognitive affective syndrome. Simultaneously, divergence between the factor structures reveals groupings of symptoms which would allow alternative clinically relevant neuropsychiatric constructs to be measured. For instance, the six-factor model separates symptoms of anxiety and of depression into distinct clusters, contrasting with the *emotional control* domain of the *a priori* model.

The comparative approach employed in this study has allowed us to carefully evaluate the internal structure of the CNRS-2. The emergence of alternative factor structures, differing from the original *a priori* five-domain structure in both factor and fragmentation in interpretable factors, demonstrates phenomenological overlap of neuropsychiatric concepts (33). We present these differing structures as complementary perspectives on the internal structure of the scale. Neuropsychiatric symptoms may cooccur, for example overlap between symptoms of anxiety and ADHD, or impulsivity and difficulties with social cognition, (34–40) or may have a shared neurobiological mechanism, for instance *F6* of the EFA-derived solution comprises symptoms that may be underpinned by a shared disruption in affective, sensory, and cognitive control mechanisms. The alternative fragmentation of the CNRS-2 items between the six factors of the EFA-derived solution offers further rationale for the organization of the *a priori* domains and overshoot–undershoot dipoles and helps explain the overlapping constructs measured within the scale. For instance, the incorporation of anxiety and depression into the same *emotional control* domain in the five-domain model reflects their high comorbidity in clinically presenting cohorts (34, 41). The additional grouping of the items between the overshoot–undershoot dipoles in the *emotional contr*ol domain, however, as with the other domains, makes it possible to view these two constructs independently.

Throughout the analyses performed in this study, overlap and interrelation between symptoms and concepts measured by the CNRS-2 was apparent, highlighting an inherent challenge in neuropsychiatry of separating psychiatric features into discrete constructs. This notwithstanding, the five-domain *a priori* model derived from clinical insight (5) is substantiated here by empirical data. The CNRS-2 with its five domains captures the complexity of neuropsychiatric phenomenology, while remaining cognizant of blurred boundaries between psychiatric constructs, identifying and quantifying these manifestations in patients with disorders of the cerebellum.

## Limitations

There were limitations to this analysis that should be considered when interpreting our results. Whereas our sample size exceeded the minimum required 5 subjects per CNRS-2 item, a larger sample would improve reliability by increasing the stability and precision of the factor loading estimations. Due to this sample size, it was not feasible to perform an internal validation via CFA of our proposed factor models in a new sample of participants. We therefore consider the findings from the CFA to be supportive rather than definitive validations of the EFA-derived and *a priori* models. Replication of our findings in larger samples will enable more definitive validation of both the domain and overshoot–undershoot subdomain structures.

Our patient sample was heterogeneous, encompassing a broad range of cerebellar disease diagnoses, as well as variation in ataxia motor severity and psychiatric medication use, see Tables 1, 2. This enhances the generalizability of our findings on the CNRS-2 structure across cerebellar disease diagnosis, severity, and psychiatric medication groups. Particularly concerning the cerebellar disease types, an important next step will be to perform factor invariance analyses by subtype to assess variation in CNRS-2 domain structure across cerebellar disease etiologies. Our preliminary screenings of the CNRS-2 item data found there to be no significant effect of motor ataxia severity on item score endorsements, which replicates findings in the CNRS-2 validation study observing no association between motor severity and scale scores, see Supplementary Table S1 for demographic and clinical characteristic effects (7). However, motor severity is still an important determinant to account for in future studies of the CNRS-2 in these patient groups. Psychiatric medication use was found to have a significant association with item scores. While psychiatric treatment may have helped to improve symptoms, participants reporting medication use had on average higher CNRS-2 scores than participants who did not. Analyses did not account for the effect of psychiatric medications on CNRS-2 scores, future studies should control for this as well as other psychiatric treatment modalities, including cognitive behavioral therapy (CBT).

This study was limited to just English-speaking participants, with the majority of participants reporting residence in the United States. This may bias interpretation and endorsement of items, particularly those that are socially framed. Once translations of the CNRS-2 are validated and made available, differential analysis of the scale by country and language will become more feasible, enhancing generalizability across cultures and populations. Furthermore, participants reported relatively high levels of educational attainment, with a mean of 14.5 years (SD 4.5). Even though we did not observe any significant effects of education on CNRS-2 item endorsement, this higher average education level still may have influenced participant comprehension of more nuanced items in the scale. Evaluation of the scale in a more educationally diverse cohort would facilitate generalizability of findings to patients with lower educational levels.

The CNRS-2 items are scored on an ordinal four-point Likert scale and exhibit a non-parametric distribution. We selected appropriate analytical approaches to account for this and reported robust fit indices, however we acknowledge that the lack of normality might still have impacted the factor model estimations and statistical fit.

Some of the CNRS-2 items demonstrated weaker suitability when we evaluated their skewness and kurtosis and had lower factor loadings, *λ* ≤ 0.30, in the EFA. Unacceptable skewness and kurtosis may destabilize and reduce the reliability and power of the factor solutions, so these items could be viewed as candidates for removal from the scale to optimize the subsequent factor analysis. However, during the cognitive debrief stage of the CNRS-2 development, the feedback we received from patients and their families provided a consensus on the clinical relevance and importance of all the items included in the final version of the scale (7). Items with lower loadings reflect symptoms that are less commonly experienced (e.g., hallucinations), yet these are recognized manifestation of the CCAS in some individuals and may be especially impactful. From our clinical experience, some of the *autism spectrum* and *psychosis spectrum* items are more prevalent in children, who were not included in this analysis, and in adults in the late stages of cerebellar neurodegenerative diseases, of whom only a small number were in this cohort. We may also expect different diagnostic or etiological patient groups to experience some symptoms more than others. Recognizing the importance of capturing these symptoms, we did not remove any CNRS-2 items.

This factor analysis study focused exclusively on the self-report version of the CNRS-2. Insight can be impaired in patients with cerebellar disorders, influencing CNSRS-2 item endorsements, therefore a similar analysis of the informant-report version is needed with subsequent evaluation of the convergence of factor structures between the two versions of the scale.

## Conclusion

This data-driven factor analysis of the items comprising the CNRS-2 provided empirical support for the clinically-derived *a priori* conceptual framework that categorizes cerebellar neuropsychiatry into the five domains of attentional control, emotional control, autism spectrum, psychosis spectrum, and social skill set. Convergence between our *a priori* five-domain model and the EFA-derived six-factor solution revealed stable and interpretable clusters of symptoms. Bifactor analysis within each domain provided support for the overshoot-undershoot dichotomy, notably in the domains of *emotional control*, *autism spectrum*, and *psychosis spectrum*. These findings underscore the presence of coherent multidimensional interrelated neuropsychiatric constructs in cerebellar disease that can be identified and measured by the CNRS-2.

## Data Availability

The raw data supporting the conclusions of this article are available upon reasonable request to the corresponding author.
