# Supplementary material for: Factor analysis validates the internal structure of the Cerebellar Neuropsychiatric Rating Scale Version 2 and the five domains of cerebellar neuropsychiatry
Source: Front Neurol. 2026 May 20;17:1784525. doi: 10.3389/fneur.2026.1784525 (PMC13230212; doi:10.3389/fneur.2026.1784525)
Supplement: Supplementary file 1 [file Supplementary_file_1.docx]

**Appendix A. Mapping of CNRS-2 item numbering.**

| **Present study** | **Item** | **Published CNRS-2** |
| --- | --- | --- |
| **1** | I make careless mistakes or do not attend to details | **1** |
| **2** | I experience hallucinations, or see or hear things that others do not | **30** |
| **3** | I feel indifferent, uninterested, or unmotivated | **33** |
| **4** | I move about or fidget excessively (not because of tremor or other movement disorder) | **2** |
| **5** | I act in an immature or childlike manner, or act too young for my age | **42** |
| **6** | I am drawn to sensory experiences | **24** |
| **7** | I do not react emotionally to situations or events that would usually produce an emotional response | **34** |
| **8** | I have difficulty stopping repetitive thoughts, urges or images that cause distress | **7** |
| **9** | I communicate in a way that is illogical or difficult for others to follow | **31** |
| **10** | I feel paranoid, or am concerned or believe that I am threatened in some way | **32** |
| **11** | I experience sudden episodes of intense fear or discomfort | **12** |
| **12** | I have trouble relating or responding to another person’s feelings | **35** |
| **13** | I avoid or feel very sensitive to light touch or texture | **27** |
| **14** | I lack pleasure from activities or interactions that used to be enjoyable | **18** |
| **15** | I am overly protective or territorial about shared space and objects | **37** |
| **16** | I have difficulty adapting to social situations | **43** |
| **17** | I am easily annoyed or irritable | **38** |
| **18** | I am easily overwhelmed by sensory input | **28** |
| **19** | I get mentally stuck or have trouble shifting focus from one activity to another | **8** |
| **20** | I speak to others or behave in a way that is not appropriate to the situation | **44** |
| **21** | I feel driven to perform habits in an excessive or repetitive manner | **3** |
| **22** | I complete tasks or behave in a rigid, inflexible way according to specific rules | **25** |
| **23** | I lack awareness of others’ personal space | **45** |
| **24** | I disobey, am non-compliant, or refuse to cooperate with instructions or requests | **39** |
| **25** | I am overly trusting of others | **46** |
| **26** | I am generally dissatisfied with life, or feel that nothing goes my way or no-one likes me | **19** |
| **27** | I act impulsively, such as interrupting others or speaking without thinking | **47** |
| **28** | I am easily distracted by non-essential or irrelevant information | **4** |
| **29** | I am passive and rely on others to take the lead | **48** |
| **30** | I experience anxiety, or have worries and feelings of nervousness | **13** |
| **31** | I continuously or repeatedly think about the same topics (including my own emotional or physical state) | **9** |
| **32** | I make repetitive movements or sounds that follow a particular pattern | **26** |
| **33** | I have difficulty maintaining focus or concentrating | **5** |
| **34** | I have negative attitudes or pessimism about the future | **20** |
| **35** | I feel unaware of social cues | **49** |
| **36** | I have difficulty controlling anger | **40** |
| **37** | I am sensitive to people entering my personal space | **41** |
| **38** | I cry and/or laugh in an exaggerated or inappropriate way or without an apparent trigger | **14** |
| **39** | I repeat myself (e.g., saying a word or phrase, making a gesture or behavior) over and over | **10** |
| **40** | I experience rapid changes in emotion that are more intense than usual | **15** |
| **41** | I feel overly active or compelled to do things and can be difficult for others to keep up with | **6** |
| **42** | I feel very sad or depressed | **21** |
| **43** | I feel distant or disengaged around others | **36** |
| **44** | I experience feelings of hopelessness | **22** |
| **45** | I have emotional feelings that do not match the situation | **16** |
| **46** | I become easily frustrated or emotional, out of proportion with the situation | **17** |
| **47** | I struggle to grasp the importance of, or understanding the emotional meaning of, a situation or event | **23** |
| **48** | I feel overwhelmed by large amounts of information or multiple tasks | **11** |
| **49** | I take things too literally | **29** |
| **50** | I have difficulty understanding the intended meaning of conversations or statements | **50** |

**Appendix B. EFA-derived six-factor CNRS-2 structure.**

|  | **Item** |  | **CFA λ** |
| --- | --- | --- | --- |
| **F1** | **9** | I communicate in a way that is illogical or difficult to follow | 0.653 |
|  | **11** | I experience sudden episodes of intense fear or discomfort | 0.659 |
|  | **15** | I am overly protective or territorial about shared space and objects | 0.710 |
|  | **16** | I have difficulty adapting to social situations | 0.781 |
|  | **17** | I am easily annoyed or irritable | 0.739 |
|  | **18** | I am easily overwhelmed by sensory input | 0.722 |
|  | **19** | I get mentally stuck or have trouble shifting focus from one activity to another | 0.848 |
|  | **22** | I complete tasks/behave in a rigid, inflexible way according to specific rules | 0.729 |
|  | **28** | I am easily distracted by non-essential or irrelevant information | 0.763 |
|  | **29** | I am passive and rely on others to take the lead | 0.630 |
|  | **30** | I experience anxiety, or have worries and feelings of nervousness | 0.746 |
|  | **31** | I continuously or repeatedly think about the same topics (including my own emotional or physical state) | 0.776 |
|  | **37** | I am sensitive to people entering my personal space | 0.558 |
|  | **48** | I feel overwhelmed by large amounts of information or multiple tasks | 0.773 |
|  | **49** | I take things too literally | 0.749 |
| **F2** | **3** | I feel indifferent, uninterested, or unmotivated | 0.805 |
|  | **14** | I lack pleasure from activities or interactions that used to be enjoyable | 0.773 |
|  | **26** | I am generally dissatisfied with life, or feel that nothing goes my way or no-one likes me | 0.886 |
|  | **34** | I have negative attitudes and pessimism about the future | 0.729 |
|  | **42** | I feel very sad or depressed | 0.879 |
|  | **43** | I feel distant or disengaged around others | 0.942 |
|  | **44** | I experiences feelings of hopelessness | 0.864 |
| **F3** | **20** | I speak to others or behave in a way that is not appropriate to the situation | 0.820 |
|  | **23** | I lack awareness of others’ personal space | 0.775 |
|  | **24** | I disobey, am non-compliant, or refuse to cooperate with instructions or requests | 0.665 |
|  | **25** | I am overly trusting of others | 0.590 |
|  | **27** | I act impulsively, such as interrupting others or speaking without thinking | 0.737 |
|  | **35** | I feel unaware of social cues | 0.827 |
|  | **36** | I have difficulty controlling anger | 0.663 |
|  | **40** | I experience rapid changes in emotion that are more intense than usual | 0.765 |
|  | **41** | I feel overly active or compelled to do things, and can be difficult for others to keep up with | 0.603 |
|  | **45** | I have emotional feelings that do not match the situation | 0.794 |
|  | **46** | I become easily frustrated or emotional, out of proportion with the situation | 0.749 |
|  | **47** | I struggle to grasp the importance of, or understanding the emotional meaning of, a situation or event | 0.867 |
|  | **50** | I have difficulty understanding the intended meaning of conversations or statements | 0.846 |
| **F4** | **2** | I experience hallucinations, or see or hear things that others do not | 0.596 |
|  | **4** | I move about or fidget excessively (not because of tremor or other movement disorder) | 0.702 |
|  | **21** | I feel driven to perform habits in an excessive or repetitive manner | 0.857 |
|  | **32** | I make repetitive movements or sounds that follow a particular pattern | 0.664 |
|  | **39** | I repeat myself (e.g., saying a word or phrase, making a gesture or behavior) over and over | 0.804 |
| **F5** | **1** | I make careless mistakes or do not attend to details | 0.729 |
|  | **7** | I do not react emotionally to situations or events that would usually produce an emotional response | 0.636 |
|  | **12** | I have trouble relating or responding to another person’s feelings | 0.804 |
|  | **33** | I have difficulty maintaining focus or concentrating | 0.796 |
| **F6** | **5** | I act in an immature or childlike manner, or act too young for my age | 0.743 |
|  | **6** | I am drawn to sensory experiences | 0.632 |
|  | **8** | I have difficulty stopping repetitive thoughts, urges or images that cause distress | 0.841 |
|  | **10** | I feel paranoid, or am concerned or believe that I am threatened in some way | 0.728 |
|  | **13** | I avoid or am overly sensitive to light touch or texture | 0.666 |
|  | **38** | I cry and/or laugh in an exaggerated or inappropriate way or without an apparent trigger | 0.591 |

**Appendix C. Hypothesized five-domain CNRS-2 structure.**

|  | **Item** |  | **CFA λ** |
| --- | --- | --- | --- |
| **AC** | **1** | I make careless mistakes or do not attend to details | 0.658 |
|  | **4** | I move about or fidget excessively (not because of tremor or other movement disorder) | 0.588 |
|  | **8** | I have difficulty stopping repetitive thoughts, urges or images that cause distress | 0.792 |
|  | **19** | I get mentally stuck or have trouble shifting focus from one activity to another | 0.857 |
|  | **21** | I feel driven to perform habits in an excessive or repetitive manner | 0.739 |
|  | **28** | I am easily distracted by non-essential or irrelevant information | 0.773 |
|  | **31** | I continuously or repeatedly think about the same topics (including my own emotional or physical state) | 0.783 |
|  | **33** | I have difficulty maintaining focus or concentrating | 0.727 |
|  | **39** | I repeat myself (e.g., saying a word or phrase, making a gesture or behavior) over and over | 0.699 |
|  | **41** | I feel overly active or compelled to do things and can be difficult for others to keep up with | 0.595 |
|  | **48** | I feel overwhelmed by large amounts of information or multiple tasks | 0.784 |
| **EC** | **11** | I experience sudden episodes of intense fear or discomfort | 0.692 |
|  | **14** | I lack pleasure from activities or interactions that used to be enjoyable | 0.700 |
|  | **26** | I am generally dissatisfied with life, or feel that nothing goes my way or no-one likes me | 0.828 |
|  | **30** | I experience anxiety, or have worries and feelings of nervousness | 0.774 |
|  | **34** | I have negative attitudes or pessimism about the future | 0.666 |
|  | **38** | I cry and/or laugh in an exaggerated or inappropriate way or without an apparent trigger | 0.572 |
|  | **40** | I experience rapid changes in emotion that are more intense than usual | 0.772 |
|  | **42** | I feel very sad or depressed | 0.815 |
|  | **44** | I experience feelings of hopelessness | 0.820 |
|  | **45** | I have emotional feelings that do not match the situation | 0.806 |
|  | **46** | I become easily frustrated or emotional, out of proportion with the situation | 0.763 |
|  | **47** | I struggle to grasp the importance of, or understanding the emotional meaning of, a situation or event | 0.903 |
| **AS** | **6** | I am drawn to sensory experiences | 0.598 |
|  | **13** | I avoid or feel very sensitive to light touch or texture | 0.631 |
|  | **18** | I am easily overwhelmed by sensory input | 0.725 |
|  | **22** | I complete tasks or behave in a rigid, inflexible way according to specific rules | 0.736 |
|  | **32** | I make repetitive movements or sounds that follow a particular pattern | 0.575 |
|  | **49** | I take things too literally | 0.757 |
| **PS** | **2** | I experience hallucinations, or see or hear things that others do not | 0.516 |
|  | **3** | I feel indifferent, uninterested, or unmotivated | 0.720 |
|  | **7** | I do not react emotionally to situations or events that would usually produce an emotional response | 0.581 |
|  | **9** | I communicate in a way that is illogical or difficult for others to follow | 0.660 |
|  | **10** | I feel paranoid, or am concerned or believe that I am threatened in some way | 0.688 |
|  | **12** | I have trouble relating or responding to another person’s feelings | 0.726 |
|  | **43** | I feel distant or disengaged around others | 0.843 |
| **SS** | **5** | I act in an immature or childlike manner, or act too young for my age | 0.699 |
|  | **15** | I am overly protective or territorial about shared space and objects | 0.711 |
|  | **16** | I have difficulty adapting to social situations | 0.781 |
|  | **17** | I am easily annoyed or irritable | 0.745 |
|  | **20** | I speak to others or behave in a way that is not appropriate to the situation | 0.811 |
|  | **23** | I lack awareness of others’ personal space | 0.757 |
|  | **24** | I disobey, am non-compliant, or refuse to cooperate with instructions or requests | 0.651 |
|  | **25** | I am overly trusting of others | 0.584 |
|  | **27** | I act impulsively, such as interrupting others or speaking without thinking | 0.728 |
|  | **29** | I am passive and rely on others to take the lead | 0.635 |
|  | **35** | I feel unaware of social cues | 0.814 |
|  | **36** | I have difficulty controlling anger | 0.653 |
|  | **37** | I am sensitive to people entering my personal space | 0.561 |
|  | **50** | I have difficulty understanding the intended meaning of conversations or statements | 0.834 |
