# Supplementary material for: Factor analysis validates the internal structure of the Cerebellar Neuropsychiatric Rating Scale Version 2 and the five domains of cerebellar neuropsychiatry
Source: Front Neurol. 2026 May 20;17:1784525. doi: 10.3389/fneur.2026.1784525 (PMC13230212; doi:10.3389/fneur.2026.1784525)
Supplement: Supplementary file 2 [file Data_sheet_1.pdf]

Supplementary Table S1. Demographic and clinical effects on CNRS-2 item scores

	**Gender ***	**Age ****	**Diagnosis *****	**Disease duration ****	**Functional stage ****	**Gait severity *****	**Education level *****	**Education duration ****	**Psychiatric medication use ***
**1**	0.896	0.784	0.631	0.994	0.061	0.853	0.291	0.396	0.003
**2**	0.999	0.315	0.615	0.994	0.857	0.948	0.243	0.121	0.328
**3**	0.662	0.149	0.763	0.994	0.130	0.647	0.444	0.390	0.007
**4**	0.785	0.016	0.154	0.908	0.333	0.853	0.444	0.363	< 0.001
**5**	0.662	0.016	0.631	0.908	0.455	0.647	0.291	0.363	0.048
**6**	0.448	< 0.001	0.053	0.908	0.962	0.744	0.540	0.363	0.026
**7**	0.448	0.356	0.631	0.994	0.173	0.647	0.291	0.390	0.047
**8**	0.994	0.001	0.526	0.994	0.857	0.647	0.243	0.462	0.003
**9**	0.993	0.209	0.533	0.908	0.588	0.948	0.160	0.390	0.013
**10**	0.896	0.010	0.211	0.994	0.654	0.948	0.372	0.887	0.093
**11**	0.448	0.019	0.631	0.908	0.962	0.744	0.531	0.927	0.001
**12**	0.038	0.226	0.631	0.994	0.695	0.647	0.259	0.363	0.005
**13**	0.662	0.052	0.165	0.994	0.282	0.948	0.243	0.363	0.015
**14**	0.723	0.876	0.631	0.994	0.130	0.744	0.931	0.887	0.001
**15**	0.662	0.016	0.327	0.908	0.857	0.948	0.614	0.652	0.006
**16**	0.662	0.198	0.631	0.994	0.158	0.948	0.372	0.363	< 0.001
**17**	0.662	0.046	0.763	0.908	0.824	0.948	0.372	0.363	0.001
**18**	0.662	0.141	0.211	0.908	0.533	0.948	0.583	0.927	< 0.001
**19**	0.974	0.010	0.631	0.994	0.839	0.948	0.328	0.693	< 0.001
**20**	0.448	0.046	0.631	0.994	0.744	0.948	0.256	0.671	0.131
**21**	0.993	0.016	0.631	0.908	0.962	0.688	0.469	0.396	0.001
**22**	0.723	0.218	0.675	0.908	0.158	0.647	0.482	0.418	0.012
**23**	0.352	0.421	0.703	0.908	0.857	0.948	0.623	0.488	0.114
**24**	0.038	0.856	0.631	0.908	0.992	0.853	0.259	0.887	0.490
**25**	0.662	0.356	0.503	0.681	0.701	0.948	0.243	0.995	0.178
**26**	0.572	0.033	0.631	0.994	0.192	0.647	0.372	0.488	0.003
**27**	0.352	0.120	0.615	0.862	0.857	0.744	0.482	0.887	0.004
**28**	0.896	0.397	0.631	0.994	0.701	0.647	0.713	0.927	0.004
**29**	0.993	0.429	0.563	0.994	0.588	0.948	0.243	0.887	0.004
**30**	0.038	0.024	0.687	0.908	0.744	0.948	0.372	0.578	< 0.001
**31**	0.662	0.017	0.778	0.994	0.801	0.948	0.291	0.363	< 0.001
**32**	0.974	0.715	0.631	0.908	0.158	0.647	0.372	0.390	0.019
**33**	0.448	0.984	0.745	0.994	0.495	0.948	0.451	0.914	< 0.001
**34**	0.216	0.859	0.687	0.994	0.455	0.948	0.259	0.837	0.104
**35**	0.482	0.452	0.631	0.681	0.455	0.973	0.259	0.927	0.033
**36**	0.216	0.173	0.731	0.994	0.857	0.972	0.189	0.396	0.072
**37**	0.993	0.856	0.533	0.908	0.744	0.973	0.741	0.914	0.055
**38**	0.994	0.441	0.631	0.994	0.254	0.948	0.259	0.363	0.486
**39**	0.896	0.110	0.631	0.994	0.962	0.647	0.259	0.789	0.003
**40**	0.993	0.028	0.563	0.994	0.183	0.948	0.555	0.312	< 0.001
**41**	0.662	0.076	0.978	0.994	0.857	0.948	0.350	0.914	0.136
**42**	0.286	0.206	0.675	0.994	0.158	0.853	0.243	0.363	0.001
**43**	0.896	0.046	0.763	0.908	0.282	0.948	0.328	0.363	< 0.001
**44**	0.216	0.076	0.778	0.994	0.183	0.688	0.372	0.419	0.001
**45**	0.448	0.784	0.631	0.994	0.192	0.948	0.259	0.363	0.001
**46**	0.896	0.614	0.731	0.994	0.455	0.948	0.189	0.363	< 0.001
**47**	0.038	0.900	0.325	0.908	0.268	0.948	0.160	0.396	0.001
**48**	0.448	0.237	0.631	0.994	0.273	0.948	0.372	0.847	< 0.001
**49**	0.999	0.209	0.631	0.994	0.882	0.948	0.417	0.796	0.014
**50**	0.352	0.429	0.533	0.994	0.158	0.948	0.384	0.671	0.001

**Wilcoxon test; **Spearman correlations; ***Kruskal-Wallis test. Adjusted p-values reported.*

Supplementary Table S2. CNRS-2 item Kaiser-Meyer-Olkin (KMO) values

	KMO value
**1**	0.95
**2**	0.87
**3**	0.94
**4**	0.91
**5**	0.94
**6**	0.92
**7**	0.91
**8**	0.97
**9**	0.96
**10**	0.88
**11**	0.92
**12**	0.92
**13**	0.92
**14**	0.94
**15**	0.94
**16**	0.95
**17**	0.94
**18**	0.95
**19**	0.97
**20**	0.96
**21**	0.92
**22**	0.93
**23**	0.94
**24**	0.92
**25**	0.90
**26**	0.95
**27**	0.96
**28**	0.95
**29**	0.94
**30**	0.93
**31**	0.93
**32**	0.80
**33**	0.93
**34**	0.93
**35**	0.95
**36**	0.92
**37**	0.93
**38**	0.89
**39**	0.93
**40**	0.94
**41**	0.91
**42**	0.93
**43**	0.97
**44**	0.93
**45**	0.93
**46**	0.95
**47**	0.94
**48**	0.95
**49**	0.96
**50**	0.96

Supplementary Table S3. CNRS-2 eigenvalues

	CNRS-2 eigenvalue	% variance	Cumulative variance	Random eigenvalue
**1**	17.790	100.0	100.0	0.990
**2**	2.761	15.5	115.5	0.854
**3**	1.179	6.6	122.1	0.793
**4**	1.056	5.9	128.1	0.731
**5**	1.008	5.7	133.7	0.671
**6**	0.787	4.4	138.2	0.614
**7**	0.701	3.9	142.1	0.566
**8**	0.524	2.9	145.1	0.517
**9**	0.450	2.5	147.6	0.487
**10**	0.400	2.2	149.8	0.446
**11**	0.213	1.2	151.0	0.404
**12**	0.175	1.0	152.0	0.371
**13**	0.151	0.8	152.9	0.328
**14**	0.123	0.7	153.6	0.292
**15**	0.086	0.5	154.0	0.263
**16**	0.053	0.3	154.3	0.231
**17**	0.018	0.1	154.4	0.201
**18**	0.000	0.0	154.4	0.173
**19**	−0.031	−0.2	154.3	0.135
**20**	−0.067	−0.4	153.9	0.110
**21**	−0.068	−0.4	153.5	0.085
**22**	−0.092	−0.5	153.0	0.055
**23**	−0.106	−0.6	152.4	0.030
**24**	−0.138	−0.8	151.6	−0.001
**25**	−0.165	−0.9	150.7	−0.029
**26**	−0.176	−1.0	149.7	−0.053
**27**	−0.195	−1.1	148.6	−0.078
**28**	−0.211	−1.2	147.4	−0.100
**29**	−0.237	−1.3	146.1	−0.125
**30**	−0.242	−1.4	144.7	−0.149
**31**	−0.250	−1.4	143.3	−0.171
**32**	−0.260	−1.5	141.9	−0.200
**33**	−0.290	−1.6	140.2	−0.220
**34**	−0.307	−1.7	138.5	−0.242
**35**	−0.331	−1.9	136.6	−0.267
**36**	−0.338	−1.9	134.7	−0.291
**37**	−0.355	−2.0	132.7	−0.317
**38**	−0.366	−2.1	130.7	−0.337
**39**	−0.386	−2.2	128.5	−0.359
**40**	−0.394	−2.2	126.3	−0.379
**41**	−0.401	−2.3	124.1	−0.407
**42**	−0.416	−2.3	121.7	−0.421
**43**	−0.419	−2.4	119.4	−0.444
**44**	−0.438	−2.5	116.9	−0.465
**45**	−0.471	−2.6	114.3	−0.486
**46**	−0.477	−2.7	111.6	−0.510
**47**	−0.489	−2.8	108.8	−0.534
**48**	−0.500	−2.8	106.0	−0.559
**49**	−0.511	−2.9	103.1	−0.587
**50**	−0.558	−3.1	100.0	−0.621

Supplementary Table S4. Five-factor EFA loadings

	F1	F2	F3	F4	F5	h^2	u^2
**1**	0.24		0.27			0.45	0.55
**2**				0.39		0.23	0.77
**3**		0.68				0.67	0.33
**4**				0.57		0.47	0.53
**5**	0.52			0.30		0.43	0.57
**6**	0.35			0.34		0.33	0.67
**7**	0.51					0.40	0.60
**8**			0.30			0.53	0.47
**9**			0.31			0.37	0.63
**10**	0.29				0.27	0.31	0.69
**11**		0.34	0.31			0.41	0.59
**12**	0.52					0.56	0.44
**13**			0.23			0.25	0.75
**14**		0.46	0.39			0.52	0.48
**15**			0.40			0.43	0.57
**16**			0.56			0.56	0.44
**17**		0.31	0.44			0.54	0.46
**18**			0.73			0.57	0.43
**19**			0.59			0.67	0.33
**20**	0.73					0.62	0.38
**21**	0.30			0.42		0.49	0.51
**22**	0.34		0.60			0.56	0.44
**23**	0.72					0.57	0.43
**24**	0.67					0.43	0.57
**25**	0.36					0.29	0.71
**26**		0.77				0.68	0.32
**27**	0.38					0.43	0.57
**28**			0.33			0.49	0.51
**29**			0.40			0.40	0.60
**30**		0.37	0.51			0.68	0.32
**31**		0.33	0.43			0.61	0.39
**32**				0.51		0.30	0.70
**33**			0.33			0.50	0.50
**34**		0.79				0.58	0.42
**35**	0.51					0.55	0.45
**36**	0.49					0.45	0.55
**37**			0.49			0.32	0.68
**38**	0.37					0.26	0.74
**39**				0.37		0.39	0.61
**40**	0.37					0.50	0.50
**41**	0.42					0.26	0.74
**42**		0.89				0.76	0.24
**43**		0.63				0.68	0.32
**44**		0.90				0.79	0.21
**45**	0.43	0.35				0.52	0.48
**46**	0.32	0.32	0.32			0.53	0.47
**47**	0.69					0.65	0.35
**48**			0.70			0.61	0.39
**49**	0.30		0.55			0.52	0.48
**50**	0.40		0.43			0.58	0.42

Abbreviations: *F1 = factor 1;* h^2 *= communality;* u^2 *= unique variance*

Supplementary Table S5. Six-factor EFA loadings

	F1	F2	F3	F4	F5	F6	h^2	u^2
**1**					0.39		0.46	0.54
**2**				0.29		0.27	0.22	0.78
**3**		0.64			0.44		0.70	0.30
**4**				0.41		0.35	0.47	0.53
**5**						0.57	0.51	0.49
**6**						0.71	0.52	0.48
**7**					0.36	0.32	0.45	0.55
**8**	0.34					0.39	0.57	0.43
**9**	0.25						0.37	0.63
**10**						0.38	0.33	0.67
**11**	0.34	0.33					0.42	0.58
**12**			0.39		0.40		0.57	0.43
**13**						0.32	0.28	0.72
**14**	0.30	0.44					0.52	0.48
**15**	0.40						0.45	0.55
**16**	0.45						0.58	0.42
**17**	0.41	0.30					0.54	0.46
**18**	0.72						0.60	0.40
**19**	0.52						0.67	0.33
**20**			0.58				0.62	0.38
**21**				0.58			0.57	0.43
**22**	0.50		0.36				0.57	0.43
**23**			0.50				0.57	0.43
**24**			0.51				0.43	0.57
**25**	0.22		0.27				0.29	0.71
**26**		0.74					0.67	0.33
**27**			0.25			0.22	0.43	0.57
**28**	0.26			0.22	0.20		0.49	0.51
**29**	0.29			0.24	0.27		0.41	0.59
**30**	0.48	0.38					0.67	0.33
**31**	0.43	0.33					0.61	0.39
**32**				0.59			0.36	0.64
**33**					0.34		0.50	0.50
**34**		0.77					0.59	0.41
**35**			0.45				0.58	0.42
**36**	0.30		0.48				0.45	0.55
**37**	0.53						0.36	0.64
**38**						0.32	0.27	0.73
**39**				0.58			0.49	0.51
**40**		0.30	0.35				0.51	0.49
**41**			0.34				0.26	0.74
**42**		0.88					0.76	0.24
**43**		0.65					0.72	0.28
**44**		0.91					0.80	0.20
**45**		0.39	0.44				0.56	0.44
**46**	0.31	0.33	0.38				0.53	0.47
**47**			0.63				0.71	0.29
**48**	0.61						0.61	0.39
**49**	0.48		0.31				0.52	0.48
**50**	0.31		0.40	0.30			0.62	0.38

Supplementary Table S6. Seven-factor EFA loadings

	F1	F2	F3	F4	F5	F6	F7	h^2	u^2
**1**	0.52							0.49	0.51
**2**				0.29		0.26		0.22	0.78
**3**		0.62			0.37			0.70	0.30
**4**				0.39		0.30		0.46	0.54
**5**						0.54		0.50	0.50
**6**						0.69		0.53	0.47
**7**					0.53			0.51	0.49
**8**						0.36		0.57	0.43
**9**	0.28							0.36	0.64
**10**						0.37		0.33	0.67
**11**		0.35					0.37	0.44	0.56
**12**					0.60			0.67	0.33
**13**							0.32	0.34	0.66
**14**		0.44						0.56	0.44
**15**							0.48	0.52	0.48
**16**							0.36	0.63	0.37
**17**							0.31	0.54	0.46
**18**	0.38						0.48	0.60	0.40
**19**	0.48							0.68	0.32
**20**			0.51					0.62	0.38
**21**				0.52				0.56	0.44
**22**			0.30				0.40	0.59	0.41
**23**			0.41					0.57	0.43
**24**			0.41					0.43	0.57
**25**			0.24					0.29	0.71
**26**		0.74						0.68	0.32
**27**	0.42							0.50	0.50
**28**	0.62							0.60	0.40
**29**	0.48							0.42	0.58
**30**		0.38					0.37	0.67	0.33
**31**		0.33						0.62	0.38
**32**				0.67				0.44	0.56
**33**	0.58							0.55	0.45
**34**		0.76						0.59	0.41
**35**			0.42					0.58	0.42
**36**			0.40					0.45	0.55
**37**							0.57	0.41	0.59
**38**						0.32		0.26	0.74
**39**				0.54				0.49	0.51
**40**		0.30	0.38					0.52	0.48
**41**			0.29				0.23	0.30	0.70
**42**		0.87						0.76	0.24
**43**		0.64						0.75	0.25
**44**		0.90						0.81	0.19
**45**		0.38	0.47					0.56	0.44
**46**		0.32	0.40					0.55	0.45
**47**			0.58					0.71	0.29
**48**	0.73							0.69	0.31
**49**	0.32						0.31	0.52	0.48
**50**	0.32		0.37					0.62	0.38

Supplementary Table S7. EFA-derived six-factor solution CFA loadings

	Factor 1	Factor 2	Factor 3	Factor 4	Factor 5	Factor 6
**1**	-	-	-	-	0.720	-
**2**	-	-	-	0.600	-	-
**3**	-	0.808	-	-	-	-
**4**	-	-	-	0.690	-	-
**5**	-	-	-	-	-	0.744
**6**	-	-	-	-	-	0.635
**7**	-	-	-	-	0.638	-
**8**	-	-	-	-	-	0.842
**9**	0.657	-	-	-	-	-
**10**	-	-	-	-	-	0.730
**11**	0.663	-	-	-	-	-
**12**	-	-	-	-	0.797	-
**13**	-	-	-	-	-	0.668
**14**	-	0.770	-	-	-	-
**15**	0.707	-	-	-	-	-
**16**	0.778	-	-	-	-	-
**17**	0.740	-	-	-	-	-
**18**	0.720	-	-	-	-	-
**19**	0.850	-	-	-	-	-
**20**	-	-	0.821		-	-
**21**	-	-	-	0.860	-	-
**22**	0.731	-	-	-	-	-
**23**	-	-	0.770	-	-	-
**24**	-	-	0.664	-	-	-
**25**	-	-	0.596	-	-	-
**26**	-	0.888	-	-	-	-
**27**	-	-	0.742	-	-	-
**28**	0.767	-	-	-	-	-
**29**	0.632	-	-	-	-	-
**30**	0.742	-	-	-	-	-
**31**	0.775	-	-	-	-	-
**32**	-	-	-	0.668	-	-
**33**	-	-	-	-	0.798	-
**34**	-	0.727	-	-	-	-
**35**	-	-	0.827	-	-	-
**36**	-	-	0.667	-	-	-
**37**	0.560	-	-	-	-	-
**38**	-	-	-	-	-	0.595
**39**	-	-	-	0.808	-	-
**40**	-	-	0.766	-	-	-
**41**	-	-	0.606	-	-	-
**42**	-	0.866	-	-	-	-
**43**	-	0.940	-	-	-	-
**44**	-	0.863	-	-	-	-
**45**	-	-	0.789	-	-	-
**46**	-	-	0.750	-	-	-
**47**	-	-	0.866	-	-	-
**48**	0.779	-	-	-	-	-
**49**	0.752	-	-	-	-	-
**50**	-	-	0.846	-	-	-

Supplementary Table S8. EFA-derived six-factor solution factor correlations

ρ	F1	F2	F3	F4	F5	F6
**F1**	1					
**F2**	0.746	1				
**F3**	0.882	0.650	1			
**F4**	0.769	0.574	0.769	1		
**F5**	0.845	0.622	0.837	0.736	1	
**F6**	0.871	0.640	0.858	0.826	0.794	1

ρ = spearman correlation coefficient; all $P < .001$

Supplementary Table S9. *A priori* five-domain model CFA loadings

	Attentional control	Emotional control	Autism spectrum	Psychosis spectrum	Social skill set
**1**	0.658	-	-	-	-
**2**	-	-	-	0.516	-
**3**	-	-	-	0.720	-
**4**	0.588	-	-	-	-
**5**	-	-	-	-	0.699
**6**	-	-	0.598	-	-
**7**	-	-	-	0.581	-
**8**	0.792	-	-	-	-
**9**	-	-	-	0.660	-
**10**	-	-	-	0.688	-
**11**		0.692	-	-	-
**12**	-	-	-	0.726	-
**13**	-	-	0.631	-	-
**14**	-	0.700	-	-	-
**15**	-	-	-	-	0.711
**16**	-	-	-	-	0.781
**17**	-	-	-	-	0.745
**18**	-	-	0.725	-	-
**19**	0.857	-	-	-	-
**20**	-	-	-	-	0.811
**21**	0.739	-	-	-	-
**22**	-	-	0.736	-	-
**23**	-	-	-	-	0.757
**24**	-	-	-	-	0.651
**25**	-	-	-	-	0.584
**26**	-	0.828	-	-	-
**27**	-	-	-	-	0.728
**28**	0.773	-	-	-	-
**29**	-	-	-	-	0.635
**30**	-	0.774	-	-	-
**31**	0.783	-	-	-	-
**32**	-	-	0.575	-	-
**33**	0.727	-	-	-	-
**34**	-	0.666	-	-	-
**35**	-	-	-	-	0.814
**36**	-	-	-	-	0.653
**37**	-	-	-	-	0.561
**38**	-	0.572	-	-	-
**39**	0.699	-	-	-	-
**40**	-	0.772	-	-	-
**41**	0.595	-	-	-	-
**42**	-	0.815	-	-	-
**43**	-	-	-	0.843	-
**44**	-	0.820	-	-	-
**45**	-	0.806	-	-	-
**46**	-	0.763	-	-	-
**47**	-	0.903	-	-	-
**48**	0.784	-	-	-	-
**49**	-	-	0.757	-	-
**50**	-	-	-	-	0.834

Supplementary Table S10. *a priori* five domain model domain correlations

ρ	Attentional control	Emotional control	Autism spectrum	Psychosis spectrum	Social skill set
**Attentional control**	1				
**Emotional control**	0.809	1			
**Autism spectrum**	0.974	0.768	1		
**Psychosis spectrum**	0.891	0.912	0.862	1	
**Social skill set**	0.905	0.805	0.967	0.907	1

ρ = spearman correlation coefficient; all *P < .001*

Supplementary Table S11. *a priori* five-domain subdomain CFA fit indices

	Attentional control	Emotional control	Autism spectrum	Psychosis spectrum	Social skill set
**χ^2(df)**	120.48 (43)	315.21 (53)	28.30 (8)	56.45 (13)	222.97 (76)
**CFI**	0.976	0.943	0.963	0.953	0.958
**TLI**	0.970	0.929	0.930	0.924	0.950
**RMSEA**	0.081 [0.064–0.098]	0.134 [0.120–0.149]	0.096 [0.059–0.136]	0.110 [0.082–0.141]	0.084 [0.071–0.097]
**SRMR**	0.060	0.092	0.061	0.063	0.069

Abbreviations: *χ^2 = Chi-squared; df = degrees of freedom; CFI = comparative fit index; TLI = Tucker-Lewis Index; RMSEA = root mean square error of approximation; SRMR = standardized root mean square residual.*

Supplementary Table S12. *Attentional control* (AC) subdomain CFA loadings

AC Item	Overshoot	Undershoot
**1**	0.689	-
**4**	0.586	-
**8**	-	0.783
**19**	-	0.875
**21**	0.751	-
**28**	0.818	-
**31**	-	0.731
**33**	0.796	-
**39**	-	0.705
**41**	0.498	-
**48**	-	0.802

Supplementary Table S13. *Emotional control* (EC) subdomain CFA loadings

EC Item	Overshoot	Undershoot
**11**	0.743	-
**14**	-	0.645
**26**	-	0.876
**30**	0.795	-
**34**	-	0.778
**38**	0.534	-
**40**	0.772	-
**42**	-	0.903
**44**	-	0.915
**45**	0.846	-
**46**	0.781	-
**47**	-	0.652

Supplementary Table S14. *Autism spectrum* (AS) subdomain CFA loadings

AS Item	Overshoot	Undershoot
**6**	0.567	-
**13**	-	0.673
**18**	-	0.677
**22**	0.725	-
**32**	0.533	-
**49**	-	0.737

Supplementary Table S15. *Psychosis spectrum* (PS) subdomain CFA loadings

PS Item	Overshoot	Undershoot
**2**	0.598	-
**3**	-	0.743
**7**	-	0.701
**9**	0.719	-
**10**	0.612	-
**12**	-	0.799
**43**	-	0.763

Supplementary Table S16. *Social skill set* (SS) subdomain CFA loadings

SS Item	Overshoot	Undershoot
**5**	-	0.693
**15**	0.749	-
**16**	-	0.734
**17**	0.782	-
**20**	-	0.845
**23**	-	0.810
**24**	0.745	-
**25**	-	0.630
**27**	-	0.731
**29**	-	0.588
**35**	-	0.834
**36**	0.746	-
**37**	0.587	-
**50**	-	0.836

Supplementary Table S17. *a priori* five-domain subdomain CFA reliability

		Cronbach α	AVE
**Attentional control**	**Overshoot**	0.82	0.49
	**Undershoot**	0.86	0.61
**Emotional control**	**Overshoot**	0.87	0.57
	**Undershoot**	0.93	0.65
**Autism spectrum**	**Overshoot**	0.54	0.38
	**Undershoot**	0.67	0.49
**Psychosis spectrum**	**Overshoot**	0.56	0.42
	**Undershoot**	0.82	0.57
**Social skill set**	**Overshoot**	0.81	0.53
	**Undershoot**	0.90	0.56

Abbreviation: *AVE = average variance extracted*

Supplementary Figure S1. Path diagram of five-factor EFA solution

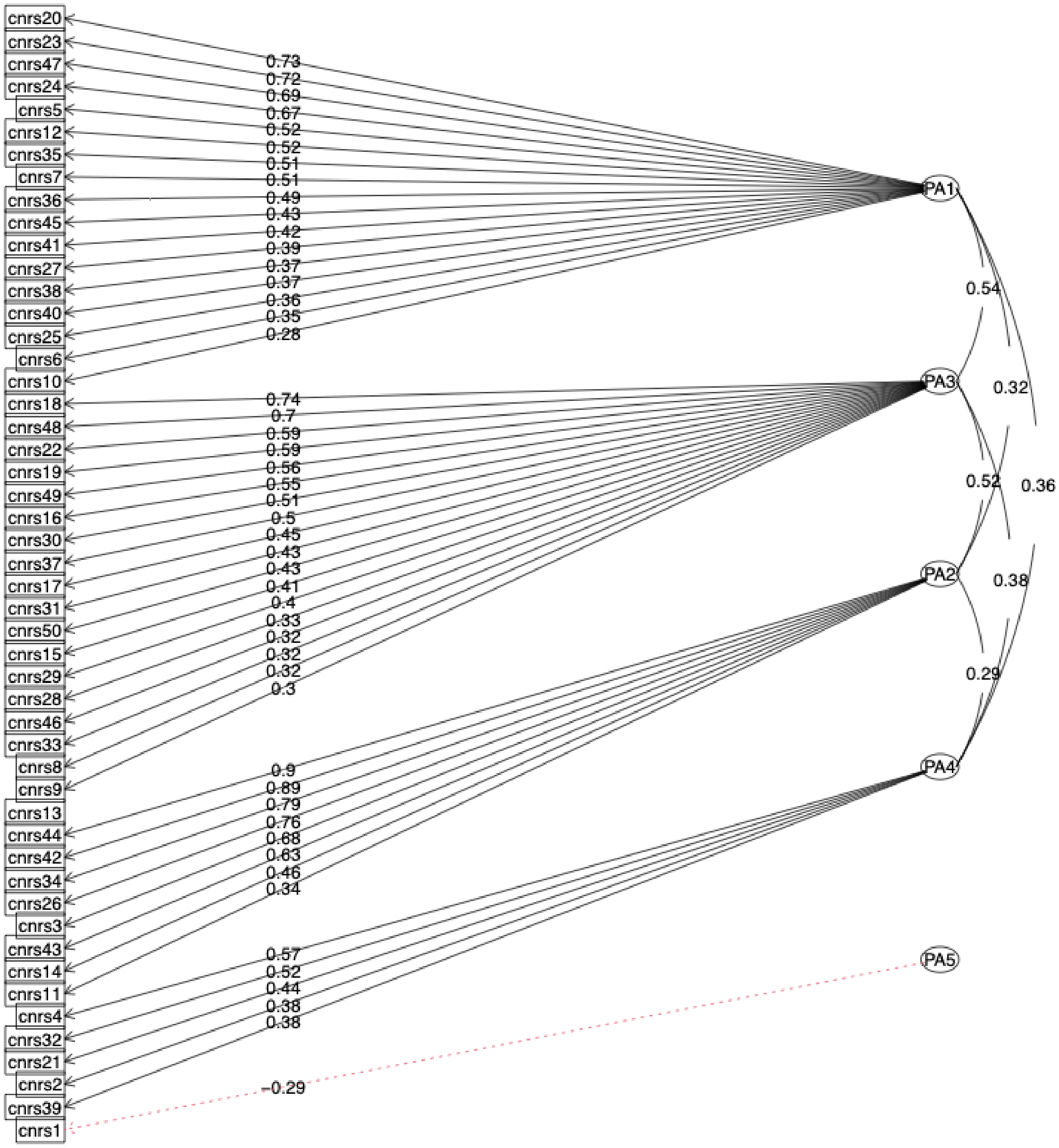

CNRS-2 items listed to the left of the diagram and the five factor on the right. EFA loadings are shown between the CNRS-2 items and the factors. Correlations between factors shown on the far-right.

Supplementary Figure S2. Path diagram of six-factor EFA solution

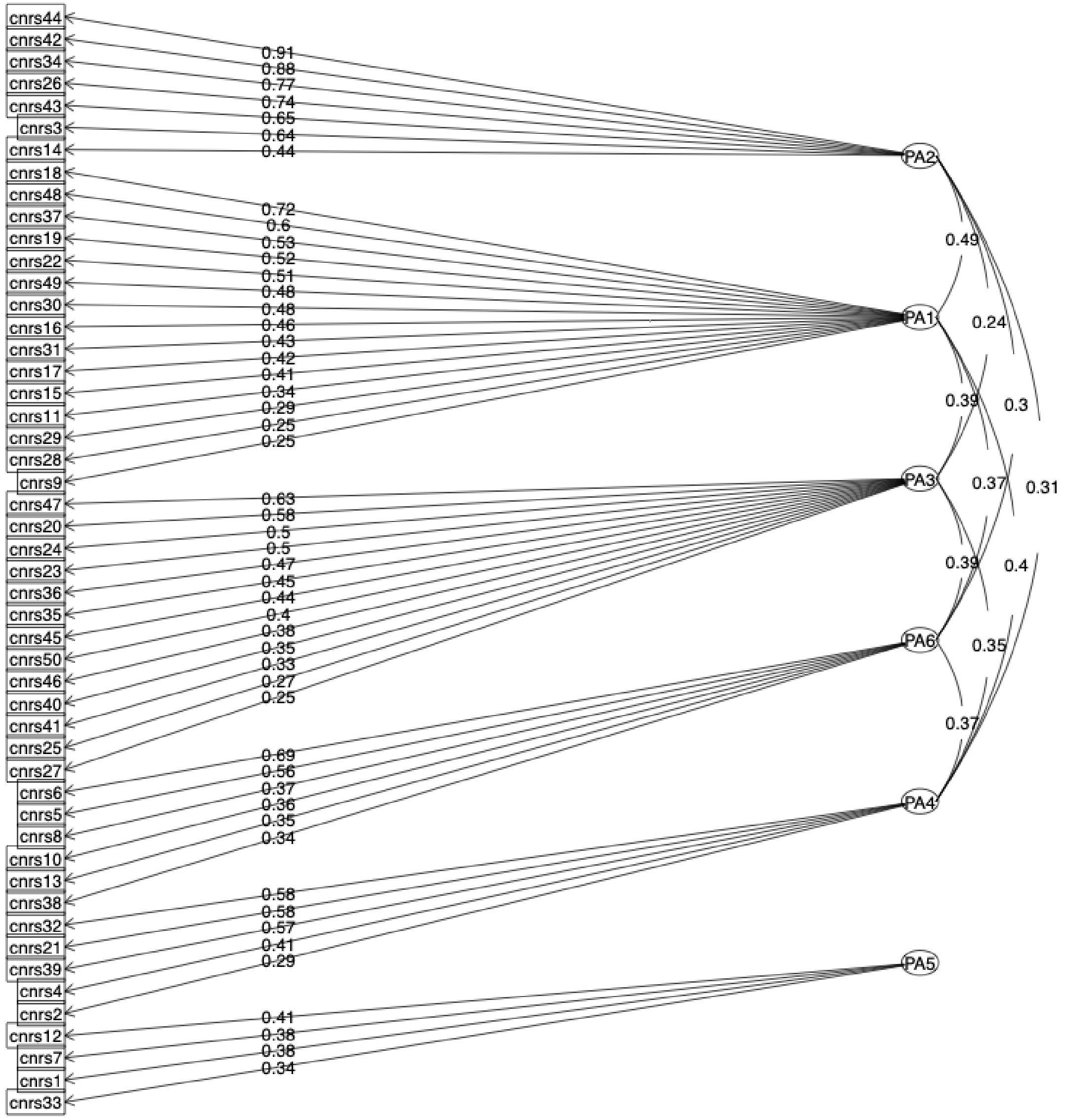

CNRS-2 items listed to the left of the diagram and the six factor on the right. EFA loadings are shown between the CNRS-2 items and the factors. Correlations between factors shown on the far-right.

Supplementary Figure S3. Path diagram of seven-factor EFA solution

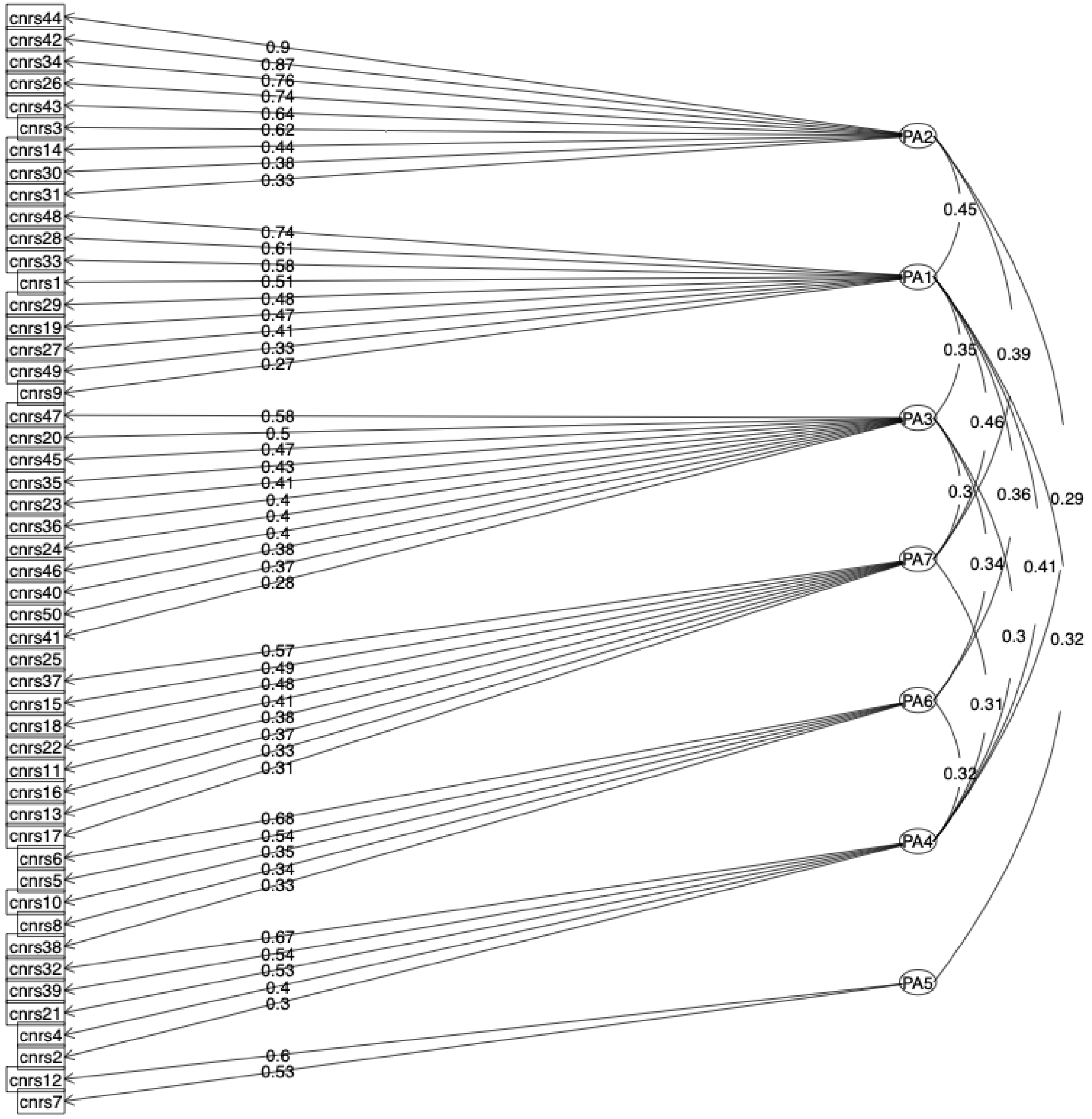

CNRS-2 items listed to the left of the diagram and the seven factor on the right. EFA loadings are shown between the CNRS-2 items and the factors. Correlations between factors shown on the far-right.
